# Longitudinal stream synoptic monitoring tracks chemicals along watershed continuums: a typology of trends

**DOI:** 10.3389/fenvs.2023.1122485

**Published:** 2023-06-09

**Authors:** Sujay S. Kaushal, Carly M. Maas, Paul M. Mayer, Tammy A. Newcomer-Johnson, Stanley B. Grant, Megan A. Rippy, Ruth R. Shatkay, Jonathan Leathers, Arthur J. Gold, Cassandra Smith, Evan C. McMullen, Shahan Haq, Rose Smith, Shuiwang Duan, Joseph Malin, Alexis Yaculak, Jenna E. Reimer, Katie Delaney Newcomb, Ashley Sides Raley, Daniel C. Collison, Joseph G. Galella, Melissa Grese, Gwendolyn Sivirichi, Thomas R. Doody, Peter Vikesland, Shantanu V. Bhide, Lauren Krauss, Madeline Daugherty, Christina Stavrou, MaKayla Etheredge, Jillian Ziegler, Andrew Kirschnick, William England, Kenneth T. Belt

**Affiliations:** 1 Department of Geology, Earth System Science Interdisciplinary Center, University of Maryland, College Park, MD, United States; 2 United States Environmental Protection Agency, Office of Research and Development, Center for Public Health and Environmental Assessment, Pacific Ecological Systems Division, Corvallis, OR, United States; 3 United States Environmental Protection Agency, Center for Environmental Measurement and Modeling, Watershed and Ecosystem Characterization Division, Cincinnati, OH, United States; 4 Occoquan Watershed Monitoring Laboratory, The Charles E. Via, Jr. Department of Civil and Environmental Engineering, Virginia Tech, Manassas, VA, United States; 5 Center for Coastal Studies, Virginia Tech, Blacksburg, VA, United States; 6 University of Maryland, College Park, MD, United States; 7 Department of Natural Resources Science, University of Rhode Island, Kingston, RI, United States; 8 AKRF, Inc., Hanover, MD, United States; 9 The Charles E. Via, Jr. Department of Civil and Environmental Engineering, Virginia Tech, Blacksburg, VA, United States; 10 Department of Geography and Environmental Systems, University of Maryland Baltimore County, Baltimore, MD, United States

**Keywords:** carbon, nutrients, metals, salt, drinking water, stream restoration, stormwater management, urban watershed continuum

## Abstract

There are challenges in monitoring and managing water quality due to spatial and temporal heterogeneity in contaminant sources, transport, and transformations. We demonstrate the importance of longitudinal stream synoptic (LSS) monitoring, which can track combinations of water quality parameters along flowpaths across space and time. Specifically, we analyze longitudinal patterns of chemical mixtures of carbon, nutrients, greenhouse gasses, salts, and metals concentrations along 10 flowpaths draining 1,765 km^2^ of the Chesapeake Bay region. These 10 longitudinal stream flowpaths are drained by watersheds experiencing either urban degradation, forest and wetland conservation, or stream and floodplain restoration. Along the 10 longitudinal stream flowpaths, we monitored over 300 total sampling sites along a combined stream length of 337 km. Synoptic monitoring along longitudinal flowpaths revealed: (1) increasing, decreasing, piecewise, or no trends and transitions in water quality with increasing distance downstream, which provide insights into water quality processes along flowpaths; (2) longitudinal trends and transitions in water quality along flowpaths can be quantified and compared using simple linear and non-linear statistical relationships with distance downstream and/or land use/land cover attributes, (3) attenuation and transformation of chemical cocktails along flowpaths depend on: spatial scales, pollution sources, and transitions in land use and management, hydrology, and restoration. We compared our LSS patterns with others from the global literature to synthesize a typology of longitudinal water quality trends and transitions in streams and rivers based on hydrological, biological, and geochemical processes. Applications of LSS monitoring along flowpaths from our results and the literature reveal: (1) if there are shifts in pollution sources, trends, and transitions along flowpaths, (2) which pollution sources can spread further downstream to sensitive receiving waters such as drinking water supplies and coastal zones, and (3) if transitions in land use, conservation, management, or restoration can attenuate downstream transport of pollution sources. Our typology of longitudinal water quality responses along flowpaths combines many observations across suites of chemicals that can follow predictable patterns based on watershed characteristics. Our typology of longitudinal water quality responses also provides a foundation for future studies, watershed assessments, evaluating watershed management and stream restoration, and comparing watershed responses to non-point and point pollution sources along streams and rivers. LSS monitoring, which integrates both spatial and temporal dimensions and considers multiple contaminants together (a chemical cocktail approach), can be a comprehensive strategy for tracking sources, fate, and transport of pollutants along stream flowpaths and making comparisons of water quality patterns across different watersheds and regions.

## 1 Introduction

The link between transport and transformation of chemicals along headwater streams and downstream receiving waters is critical for managing and restoring water quality, particularly in human-impacted watersheds (*e.g.*, Alexander et al., 2000; Helton et al., 2011; Gomez-Velez et al., 2015; Wollheim et al., 2018). For almost half a century, studies have supported the theory that natural headwaters are important for retaining and transforming chemicals transported to downstream river ecosystems (Vannote et al., 1980), but there can also be substantial transport and transformation of materials and energy along urban stream networks and river continuums (*e.g.*, Kaushal and Belt, 2012; Kaushal et al., 2014a). However, more work has focused on water quality trends over time in urban watersheds (*e.g.*, Groffman et al., 2004; Kaushal et al., 2008a; Mayer et al., 2022). Relatively lesser work has focused on monitoring changes in water quality along urban flowpaths (but see Bhatt and McDowell, 2007; Kaushal et al., 2014a; Ledford and Lautz, 2015; Cooper et al., 2014; Pennino et al., 2016a; Gabor et al., 2017; Duan et al., 2017; Jin et al., 2018). The Urban Watershed Continuum (UWC) concept proposes the integration of spatial and temporal dimensions when studying natural and engineered urban flowpaths (Kaushal and Belt, 2012; Kaushal et al., 2014a; Kaushal et al., 2023a). Here, we explore applications of the UWC concept as a practical monitoring approach for multiple contaminants through longitudinal monitoring of water quality along streams across space and time.

Many streams and rivers now receive large inputs and loads of complex chemical mixtures known as watershed ‘chemical cocktails’ (Kaushal et al., 2018; Kaushal et al., 2019; Kaushal et al., 2020; Kaushal et al., 2022). As a result, there is an imbalance between increased inputs of chemical cocktails (*e.g.*, excess organic matter, nutrient, salts, and metals) and a limited efficiency of river systems to attenuate and transform contaminants. Chemical cocktails are attenuated by dilution and in-stream biogeochemical processes (*e.g.*, ecosystem metabolism, biological uptake, retention on soil exchange sites, storage in groundwater) (*sensu* Finlay, 2011; Kaushal et al., 2014a; Beaulieu et al., 2015; Wollheim et al., 2015; Wollheim et al., 2017; Wollheim et al., 2018; Kaushal et al., 2022). Exceeding the biogeochemical and hydrological capacity of streams and floodplains for attenuating and transforming chemical cocktails can increase in-stream concentrations and downstream export of multiple chemical cocktails to receiving waters (Marti et al., 2004; Wollheim et al., 2008; Loken et al., 2018).

**FIGURE 1:**
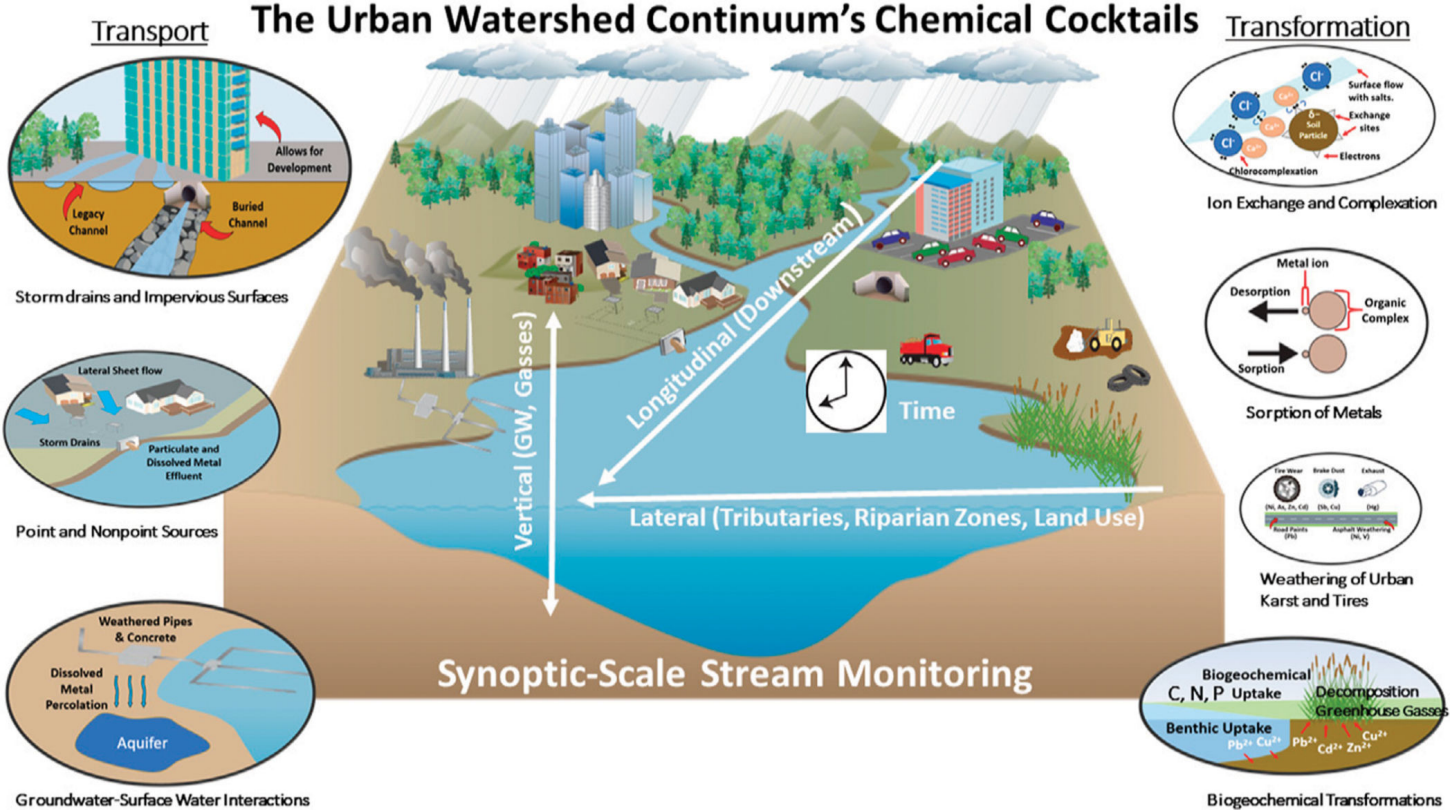
The Urban Watershed Continuum (UWC) concept proposes that materials and energy are transported and transformed along natural and engineered flowpaths across a space-time continuum (Kaushal and Belt, 2012). Longitudinal stream synoptic monitoring can help us understand how chemical cocktails are transported (left panel) and transformed (right panel) along the UWC. Chemical cocktails are novel combinations of elements, which can be studied along 4 spatial and temporal dimensions. Chemical cocktails are transported along the UWC via urban runoff, sewage inputs, or pipes. In contrast, they are transformed along the UWC by ion exchange, decomposition and dissolution, biological uptake, and mineralization.

Understanding spatial and temporal patterns in chemical cocktails along flowpaths is critical for evaluating trends and transitions in water quality (Figure 1). Along many watershed continuums, human activities have concurrently altered concentrations of organic matter, nutrients, salt ions, metals, and greenhouse gasses along flowpaths extending watershed scales (Figure 1) (Kaushal et al., 2014a; Kaushal et al., 2020; Kaushal et al., 2022; Kaushal et al., 2023b). Organic matter and nutrients from terrestrial and anthropogenic sources are transported along urban flowpaths (Carpenter et al., 1998; Mallin et al., 2006; Kaushal et al., 2011; Grant et al., 2012). These chemical cocktails are also transformed by biotic and abiotic processes (*e.g.*, microbial metabolism, photo-oxidation, photosynthesis, flocculation and sedimentation, and production of greenhouse gasses (Hall and Tank, 2003; Beaulieu et al., 2011; Finlay, 2011; Smith et al., 2017; Jin et al., 2018). In addition, salt ions overload urban watershed flowpaths from road salts, impervious surfaces, water softeners, irrigated lawns, and sewage (Kaushal et al., 2005; Cañedo-Argüelles et al., 2013; Kaushal et al., 2017; Moore et al., 2017; Bhide et al., 2021; Grant et al., 2022). Finally, flowpaths in human-impacted watersheds are enriched with metals from vehicular emissions, tire wear particles, construction materials, and other sources (Paul and Meyer, 2001; Morel et al., 2020). Monitoring watershed flowpaths across spatial and temporal dimensions has potential to inform strategies for the co-management of multiple chemicals and identifying water quality tradeoffs associated with management approaches.

Here, we demonstrate applications of longitudinal stream synoptic (LSS) monitoring of multiple contaminants along flowpaths across space and time. In general, the term “synoptic sampling” is widely used. Sometimes, synoptic means that sampling is conducted across space within a limited period of time such as across many catchment outlets (Somers et al., 2013; Hassett et al., 2018; Blaszczak et al., 2019a; Blaszczak et al., 2019b; Delesantro et al., 2022). For this paper, we define “longitudinal stream synoptic” as monitoring many points along a longitudinal stream flowpath across space and time in watersheds. For this paper, longitudinal stream synoptic surveys occur longitudinally along one stream at a time. Sometimes, our LSS monitoring surveys are repeated at the same sites along the flowpath over time to compare the role of seasonality, storms, or winter road salt events (*e.g.*, Kaushal and Belt, 2012; Kaushal et al., 2014a; Kaushal et al., 2022). We propose that LSS patterns along flowpaths represent basic units of study for evaluating trends and transitions in downstream water quality. LSS monitoring along flowpaths is relatively underutilized compared to routine monitoring, sensor deployments, random sampling surveys, and tracer studies. Many studies have focused on monitoring stream reaches at scales of a few hundreds of meters (Kaushal et al., 2023a). However, broader spatial interpretations of urban watershed patterns and processes can be complicated by heterogeneous land cover, pollution sources, and hydrologic disturbances along urban watersheds (*e.g.*, Grimm et al., 2000; Hope et al., 2005; Cadenasso et al., 2007; Sivirichi et al., 2011).

There is a need for LSS monitoring to account for spatial heterogeneity in contaminant fate and transport along watershed flowpaths (Sivirichi et al., 2011). In some cases, synoptic sampling at small watershed outlets may provide better isolation of spatial and temporal heterogeneity than LSS monitoring along flowpaths because longitudinal stream sampling can be spatially autocorrelated (*i.e.*, upstream data influences downstream data which also can result in pseudoreplication for traditional correlation analysis), and spatial heterogeneity is integrated along the major axis of temporal variation in runoff, the stream length. However, LSS monitoring along flowpaths has unique potential for: (1) assessing changes in the sources, transport, and transformation of contaminants along watershed flowpaths, (2) tracking the relative importance of management and restoration interventions along flowpaths and determining how long management and restoration signals (changes in concentrations, loads, and chemical mixtures) persist further downstream, (3) pinpointing different contaminant sources along watersheds, and (4) identifying tradeoffs in watershed restoration and management along flowpaths by analyzing combinations of chemicals (*e.g.*, decreases in some contaminants, but potential increases in others). In addition, we can learn new aspects regarding spatiotemporal heterogeneity from a hydrologic and water quality perspective. There can be significant spatial heterogeneity in the retention and transformation capacity of nutrients, salt ions, and metals on scales of meters to kilometers along urban stream flowpaths (*e.g.*, Sivirichi et al., 2011, Kaushal et al., 2014a, Newcomer Johnson et al., 2014, Pennino et al., 2016a; Pennino et al., 2016b; Smith et al., 2017, Kaushal et al., 2022). LSS monitoring integrating both spatial and temporal dimensions (Kaushal and Belt, 2012; Kaushal et al., 2014a) and analyzing multiple contaminants together (a chemical cocktail approach) (Kaushal et al., 2018; Kaushal et al., 2020) can be a comprehensive strategy for studying human-impacted watersheds and tracking sources, fate, and transport of pollutants along flowpaths.

### 1.1 Study goal: developing a typology for analyzing water quality along stream flowpaths

**FIGURE 2:**
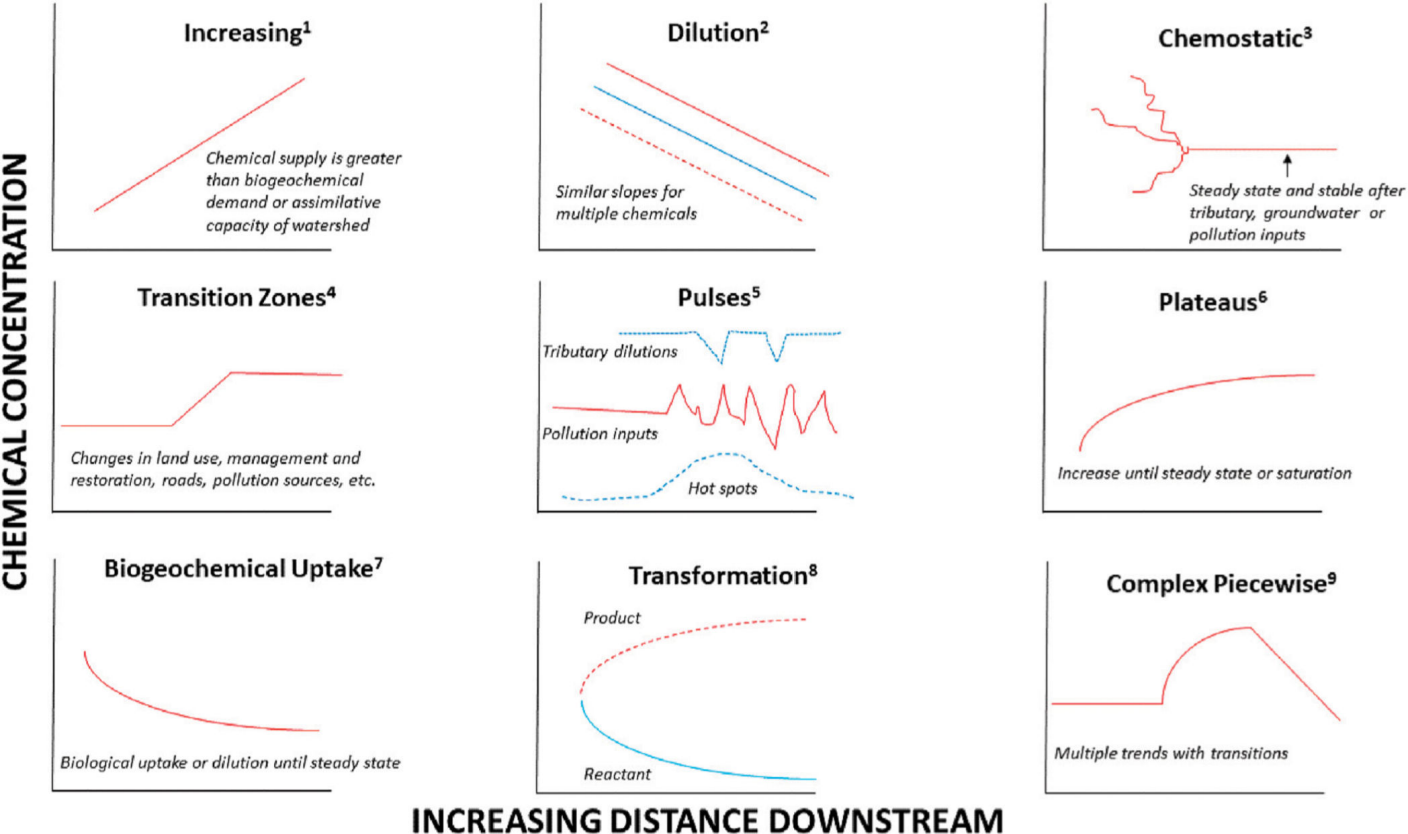
Examples of different typologies for longitudinal patterns in chemical concentrations along stream flowpaths from the literature. (1) Increasing: Previous work has documented increasing trends in chemical concentrations when watershed supply is greater than demand for biogeochemical reactions. For example, conductivity, and concentrations of Se, SO_4_^2−^ and other chemicals increase with distance downstream due to the cumulative effects of mining (Lindberg et al., 2011). In addition, concentrations of Cl^−^, PO_4_^3−^, DOC, TDN, NH_4_^+^, Na, K, Mg, Ca, SiO_2_ increased with distance downstream and increasing population density in a river in Nepal (Bhatt and McDowell, 2007). Similarly, Mg, Ca, DIC, and pH increased with distance downstream along an urban stream in Baltimore, Maryland, United States of America due to increasing watershed impervious surface cover and human-accelerated weathering (Kaushal et al., 2017). (2) Decreasing: There can also be chemical dilution along stream flowpaths and decreasing trends in chemical concentrations, particularly downstream of wastewater treatment plants (Haggard et al., 2005; Castelar et al., 2022). (3) Chemostatic: There can be chemostatic, steady state, and stable longitudinal DOC concentrations along the mainstem of a stream in Idaho, United States of America, even though in DOC concentrations in headwater tributaries were more variable. (4) Transition zones: There were abrupt transitions in Cl^−^ and NO_3_^−^ concentrations with distance downstream along a suburban restored stream in New York, United States of America, due to hydrologic storage of chemicals in a hydrologically reconnected floodplain (Ledford and Lautz, 2015). (5) Pulses: There were pulses in concentrations of Ca and Mg downstream of road and bridge crossings along streams in Baltimore, Maryland, United States of America (Sivirichi et al., 2011). Other work has documented longitudinal pulses in concentrations of CO_2_, N_2_O, and CH_4_ along the Han River in Korea in response to urban wastewater effluent (Jin et al., 2018). Tributary dilutions also contribute to conductivity pulses along the longitudinal flowpath of the Mississippi River (Loken et al., 2018). (6) Plateaus: NO_3_^−^ concentrations plateaued along a longitudinal flowpath of the Mississippi River due to biological uptake, which reached a saturation point further downstream. In addition, NO_3_^−^ and SO_4_^2−^ concentrations also plateaued downstream below a transition zone along a restored stream in New York, United States of America (Ledford and Lautz, 2015). (7) Biogeochemical Uptake: Biogeochemical uptake contributed to exponential and linear decreases in nitrate concentrations along the flowpath of a large river in Florida (Hensley et al., 2014). (8) Transformation: Biogeochemical transformations of nitrogen along longitudinal flowpaths along urban streams were revealed by inverse relationships between dissolved organic carbon (DOC) and total dissolved nitrogen (TDN) along different urban streams in Baltimore, Maryland, United States of America (Sivirichi et al., 2011). In addition, inverse relationships between Na and P, Cd, and K along longitudinal flowpaths in an urban stream after a road salt event indicated ion exchange reactions (Kaushal et al., 2023b). (9) Complex Piecewise: Many of the synoptic chemical patterns along flowpaths shown in this paper and elsewhere can be broken up into complex piecewise functions based on identifying transition points along different segments of streams and rivers.

The goal of this study was to identify, classify, and synthesize predictable patterns in water quality responses along longitudinal stream flowpaths from many observations of combinations of chemical parameters in the context of distance downstream and watershed characteristics (Figure 2). This typology of longitudinal water quality patterns along stream flowpaths can provide a foundation for future studies, watershed assessments, evaluating management and restoration efforts, and making comparisons across watershed responses. In this paper, we define typology as a system for classifying longitudinal patterns in water quality along stream flowpaths in certain categories according to how they are similar. Based on literature studies and our previous work (Figure 2; Table 1), we propose a typology with the following longitudinal patterns in chemical concentrations: (1) increasing, (2) decreasing and dilution, (3) chemostatic, (4) transition zones, (5) pulses, (6) plateaus, (7) biogeochemical uptake, (8) transformation, and (9) complex piecewise changes along flowpaths (Figure 2; Table 1). There are a growing number of examples of longitudinal synoptic studies in the literature (Table 1), which present a broader context for proposing a typology and testing patterns of responses observed in our extensive regional data sets.

Throughout this paper, we focus on the synthesis of many original data sets from LSS monitoring to test our typology and its applications in water quality studies, instead of trying to report results in a “typical” hypothesis-field collection-measurement-results-discussion type of format. The integration of multiple chemicals as chemical cocktails along flowpaths is a novel component of our typology and approach and allows tracking of pollution sources and identification of water quality tradeoffs (*e.g.*, one contaminant is retained, but another contaminant is released along flowpaths) (Kaushal et al., 2018; Kaushal et al., 2020; Kaushal et al., 2022). We use the narrative parts of this paper (and our original results and figures) to demonstrate and illustrate distinct longitudinal patterns of response observed in extensive data sets. Overall, the focus of our paper is on the synthesis of data to develop and test a typology of longitudinal water quality patterns and responses for informing water quality monitoring and management (instead of trying to report results from individual case studies).

Specifically, we compare and discuss our original data characterizing longitudinal patterns in water quality with many others from the literature to synthesize the typology of water quality trends and transitions along flowpaths. For example, we apply LSS monitoring of chemical cocktails to explore how water quality evolves as streams flow through: (1) progressively degraded areas due to urbanization, (2) restored reaches with hydrologically connected floodplains, and (3) forested conservation areas including national, regional, and local parks (natural recovery). Conservation of surrounding forested lands and wetlands, and restoration approaches involving stream-floodplain restoration can enhance attenuation and transformation of contaminants. There is a growing need for longitudinal monitoring approaches to assess whether the effects of conservation and restoration can be detected along flowpaths (Kaushal et al., 2023a).

Important mechanisms influencing attenuation and transformation include: (1) increased hydrologic residence times and contact between stream water and floodplain and hyporheic sediments, (2) ion exchange in lesser polluted soils, (3) complexation with organic matter from riparian plants and soils, (4) and biological uptake and transformation from plants and microbial communities (*e.g.*, Grant et al., 2018; Kaushal et al., 2008b, Noe and Hupp, 2009, Roley et al., 2012, Kaushal et al., 2022, Mayer et al., 2022). We propose that an improved understanding of water quality gained through LSS monitoring and a chemical cocktail approach (tracking chemical combinations across space and time) is helpful for unlocking the potential for co-management of contaminants and ecosystem recovery based on conservation and restoration practices and best management practices (BMPs) (Kaushal et al., 2022; Kaushal et al., 2023a).

## 2 Methods

### 2.1 Study design: exploring a typology of longitudinal water quality patterns

**FIGURE 3:**
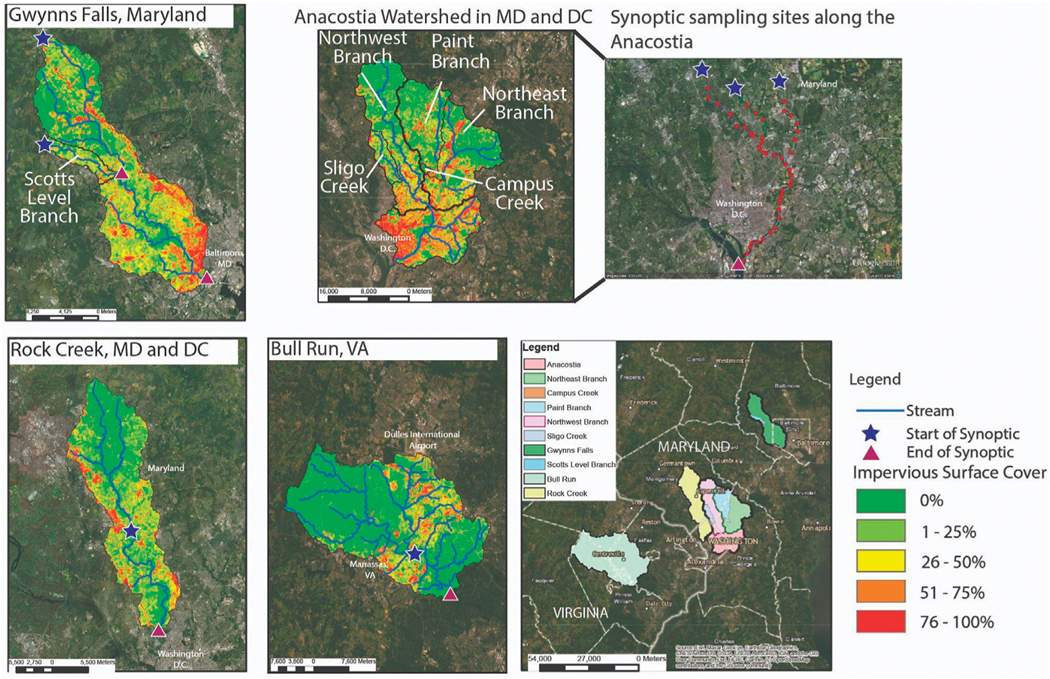
Watershed scale site maps of our 10 study watersheds are shown. The start of each longitudinal stream synoptic (LSS) monitoring is indicated by a purple star and the end of the synoptic-scale monitoring is indicated by a red triangle. One example of the high-spatial resolution monitoring is highlighted for the Anacostia watershed.

As mentioned previously, there are relatively fewer examples of longitudinal stream synoptic data compared with time-series of concentrations and fluxes from long-term monitoring sites within watersheds. Here, we investigated the sources, transport, attenuation, and transformations of chemical cocktails from data collected along 10 longitudinal stream flowpaths in the Baltimore-Washington D.C. metropolitan region (Table 2; Figure 3; Supplemental Table S1) in the Chesapeake Bay watershed within a combined 1,765 km^2^ area (Table 2; Figure 2). Detailed information on study sites and synoptic sampling surveys are in Table 2 and (Supplemental Table S1). The longitudinal stream flowpaths include data from our long-term study sites. At these sites, we have characterized and compared the hydrology, biogeochemistry, and geochemistry over decades (*e.g.*, Kaushal et al., 2005, Kaushal et al., 2008a, Kaushal et al., 2011, Kaushal and Belt, 2012; Kaushal et al., 2014a; Kaushal et al., 2014b; Kaushal et al., 2014c; Newcomer et al., 2012, Smith and Kaushal, 2015, Kaushal et al., 2019, Bhide et al., 2021, Grant et al., 2022, Kaushal et al., 2022, Kaushal et al., 2023b). These longitudinal flowpaths share similar climate and regional patterns but have: different land use characteristics (Supplemental Table S1), different management strategies, and different sources of pollution (*e.g.*, pulses of sewage effluent, urban stormwater runoff, and road salt). Urban streams and rivers in this study flow either through conservation areas (regional and national parks) and stream-floodplain restoration areas or progressively degraded urban areas (site descriptions further below). Thus, we tested the ability of LSS monitoring to provide further insights regarding sources, transport, and transformations of different chemicals at our long-term monitoring sites and to synthesize a typology of typical water quality responses to help guide future longitudinal studies.

We explored recovery in water quality at our long-term monitoring sites by comparing transport and transformation of contaminants using LSS monitoring (300 sampling sites along combined stream synoptic distances of 337 km across the 10 watershed flowpaths, in which the mainstem of the flowpath was sampled at 3–45 locations with a range in distance between sites from 0.4 to 20.85 km) (Table 2). We compared retention and release of chemical cocktails along flowpaths using statistical analysis of multiple elements or chemical cocktails. Although we conducted LSS monitoring at different times at different sites, longitudinal patterns can still be determined and quantified. In some cases, we present data from repeated LSS monitoring at our long-term monitoring sites to investigate water quality applications across seasonality and hydrologic events. Finally, we compared our longitudinal stream synoptic patterns with others from the literature to test a typology of water quality trends and transitions along flowpaths.

There are important caveats and limitations in our stream synoptic approach and the case studies that we explore throughout this paper. For example, not all stream synoptic surveys measured all the same constituents along each flowpath, not all stream synoptic surveys were subject to the same statistical tests, and not all synoptic surveys were sampled during the same season, and there were differences in spatial intervals or general time periods of different synoptic surveys. In general, the number of stream flowpaths, number of stream locations, and number of time samples were collected were based upon the following criteria: (1) long-term monitoring and background knowledge of study sites, (2) site access and safety, and (3) feasibility. Regarding long-term study sites, we have characterized the background hydrology and biogeochemistry of all of the streams mentioned in this paper including Gwynns Falls (Kaushal et al., 2008a; Kaushal et al., 2011), Campus Creek and Paint Branch (Kaushal et al., 2022; Wood et al., 2022), Scotts Level Branch (Newcomer et al., 2012; Galella et al., 2021), Sligo Creek (Galella et al., 2021, Maas et al., In Review), Bull Run (Bhide et al., 2021; Grant et al., 2022), Anacostia watershed (Kaushal et al., 2020; Kaushal et al., 2022; Kaushal et al., 2023b). In general, we typically sampled synoptic sites along every 100 m for small streams, and we sampled every 500–1,000 m along larger streams. The number of stream locations for synoptic sampling were based on site accessibility and safety. Many streams were located along bike paths where we could access sampling points quickly and frequently. At some sites we were able to conduct stream synoptic surveys routinely once per month at baseflow to characterize seasonality. In some case studies, we also sampled immediately after notable weather events, primarily during road salt applications and storms (*e.g.*, Kaushal et al., 2018; Kaushal et al., 2022). Although there were important caveats and limitations, this study aggregates and compares many results of stream synoptic surveys to observe downstream responses and synthesize a typology of water quality patterns along flowpaths based on many original case studies and the global literature (Figure 2; Table 1).

### 2.2 Site descriptions along the urban watershed continuum

#### 2.2.1 Watershed flowpaths through progressively urban degraded areas: Gwynns Falls

The Gwynns Falls watershed (39.26859, −76.62651) drains 171.5 km^2^ before entering the Patapsco River and Baltimore Harbor (Figure 3). We analyzed changes in concentrations of chemical cocktails along the mainstem of the Gwynns Falls and along major and minor tributaries. The longest flow path over which we sampled was approximately 36.5 km and located in the Piedmont physiographic province (Kaushal et al., 2014a) (Table 2). The headwaters of the Gwynns Falls watershed begin in suburban Baltimore County and then flow along urban areas of Baltimore City (Kaushal et al., 2014a). There are no point-source discharges in the Gwynns Falls watershed, but there is non-point source pollution of nutrients, salts and metals from aging sanitary infrastructure, storm drains, road salt applications, and the weathering of impervious surfaces (Kaushal et al., 2005; Kaushal et al., 2011; Kaushal and Belt, 2012; Kaushal et al., 2017). Groundwater and leaks from piped sanitary and drinking water infrastructure contribute a fraction of the flow budget in stream reaches along the Gwynns Falls (Bhaskar and Welty, 2012; Kaushal and Belt, 2012).

#### 2.2.2 UWC flowpaths through conservation and restoration areas: Anacostia River

The Anacostia River flows from suburban and urban headwaters into Anacostia National Park, where there are extensive hydrologically connected wetlands and thick riparian buffers. We conducted synoptic scale stream monitoring along six study sites within the Anacostia River watershed: (1) Anacostia River mainstem, and its main two tributaries: (2) Northeast Branch and (3) Northwest Branch. We also sampled three smaller tributaries: (4) Sligo Creek, (5) Paint Branch and (6) Campus Creek (Figure 2). The Anacostia River mainstem (38.86106, −77.01287 at the outflow) and its tributaries flow from suburban areas in Montgomery and Prince George’s Counties into Washington DC where it empties into the Potomac River (Figure 3). The Anacostia River is approximately 14 km long with a watershed area of 448 km^2^. The mainstem of the Anacostia is formed by the confluence of the Northeast Branch (5.1 km long) and Northwest Branch (34.6 km long) of the Anacostia. Anacostia National Park begins a few kilometers downstream of the confluence of the Northeast and Northwest Branches after the Anacostia River flows through Bladensburg, Maryland.

There are differences in longitudinal flowpaths of the Northeast and Northwest Branch of the Anacostia. The Northeast Branch of the Anacostia flows into progressively urban riparian zones before draining into the Anacostia mainstem. In contrast, the Northwest Branch flows into progressively urban riparian zones, but then drains into an extensive forest conservation area before emptying into the Anacostia mainstem. The watershed area of the Northeast and Northwest Branches are 194.3 and 135.5 km^2^ respectively.

A major tributary, Sligo Creek (14.6 km long), flows into the Northwest Branch. Another major tributary, Paint Branch (27.4 km long), flows into the Northeast Branch. Sligo Creek is a smaller and degraded urban watershed (19.8 km^2^) with 41.6% impervious surface cover (Galella et al., 2021) (Supplemental Table S1), which is the second highest level of impervious surface cover represented in our study besides the Gwynns Falls. Paint Branch is a tributary of the Northeast Branch of the Anacostia with a watershed area of 80.8 km^2^ and a significantly lesser percentage of impervious surface cover (31.9%) (Supplemental Table S1).

Our smallest study watershed in the Anacostia watershed was Campus Creek (~3 km long). This was a smaller stream restoration project aimed at hydrologically reconnecting floodplains with channels along headwater flowpaths. We included Campus Creek to understand the fine-scale impact of one of these smaller stream restoration projects. Campus Creek, a small tributary of Paint Branch flowing through the University of Maryland campus, originates from a storm drain and flows into a restored reach, where regenerative stormwater conveyance techniques were applied, before flowing through progressively urban areas and emptying into Paint Branch (Kaushal et al., 2022). Our detailed studies in the Anacostia River and its tributaries attempt to explore applications of LSS monitoring of flowpaths across multiple spatial and temporal scales.

#### 2.2.3 UWC flowpaths through conservation and restoration areas: Rock Creek

Rock Creek (38.90008, −77.05738 at the outfall) is a 99 km^2^ tributary of the Potomac River that originates in Montgomery County, Maryland and flows through Washington D.C., along its approximately 52.5 km long flowpath (Figure 3). The watershed flows through national and local parks and the highly urbanized metropolitan Washington D.C. area, with 20.4% forest (41 km^2^) and 32.4% impervious surfaces (65 km^2^). Our LSS monitoring started in suburban areas near Kensington, Maryland where the creek begins to flow parallel to a relatively small roadway. Rock Creek then flows underneath a major highway that encircles Washington D.C. (Capital Beltway) and flows through progressively more urban areas until it reaches the Washington D.C. border. The creek then flows for almost 6 km through Rock Creek National Park in Washington D.C., which is primarily forest and has a few roads. As it flows through Rock Creek National Park, several smaller tributaries flow into Rock Creek and there are also numerous storm drain inputs.

#### 2.2.4 UWC flowpaths through conservation and restoration areas: Scotts Level Branch

Scotts Level Branch (39.36058, −76.74620 at the outfall) is a small suburban stream that flows 8.7 km in western Baltimore County, Maryland (Newcomer et al., 2012); Scotts Level Branch is a subwatershed of the Gwynns Falls (Figure 3). The watershed area is approximately 10.4 km^2^, and it is comprised of mostly residential neighborhoods, with a forested percentage of 13.2% (~2.5 km^2^) and 39.3% impervious surfaces (~5 km^2^). The Baltimore County Department of Environmental Protection and Sustainability restored reaches of Scotts Level Branch by reconnecting the stream with its floodplain (Wood et al., 2022). Although the stream restoration occurred in different phases, most of it was completed in 2014 (Wood et al., 2022). Prior to restoration, increased streamflow from urban runoff and impervious surface cover contributed to stream channel incision, downcutting, and hydrologic disconnection between the stream and its floodplain. The goal of the floodplain reconnection was to increase stability in the stream channel, dissipate erosive force during storm events, and restore water quality. Water quality was restored by increasing hydrologic connectivity between streamwater and “hot spots” of contaminant retention and transformation in the hyporheic zones, floodplain soils, and riparian vegetation (Kaushal et al., 2008b; Newcomer et al., 2012; Mayer et al., 2022; Wood et al., 2022).

#### 2.2.5 UWC flowpaths through conservation and restoration areas: Bull Run

Bull Run (38.72412, −77.38027 at the outfall) is a 52.8 km stream that drains into the Occoquan Reservoir, which supplies 40% of the drinking water for two million people in northern Virginia (Figure 3). Bull Run drains 312 km^2^ suburban and urban land uses, and of the sites we studied has the highest percentage of forest (35.7% forest, 111 km^2^) and the lowest percentage (13%) of impervious surface cover (41 km^2^). The Upper Occoquan Service Authority (UOSA), a water reclamation facility, discharges highly treated wastewater directly into Bull Run through a management practice referred to as indirect potable reuse (Bhide et al., 2021). UOSA was built in the 1970s and is the first indirect potable reuse facility in the United States, with the goal to improve water security in the Mid-Atlantic region (Bhide et al., 2021). The combination of upstream non-point source pollution, replacement of wetlands and forests with urban areas, and direct wastewater discharges into the stream contribute to low dissolved oxygen and elevated levels of nutrients, turbidity, and other chemicals (Jones and Arciszewski, 2000; Xu et al., 2007). After receiving wastewater discharge from UOSA, Bull Run flows through a 1,568-acre regional park with extensive forest, riparian buffers and wetlands. Forest and wetlands have the capacity for natural attenuation and assimilation of contaminants; indeed, drinking water quality in the Occoquan Reservoir relies heavily on these biogeochemical processes as Bull Run flows through the Bull Regional Park (Cubas et al., 2014).

### 2.3 Longitudinal and lateral sampling of water quality along the UWC

Multiple longitudinal stream synoptic locations were located throughout each of the above watersheds to investigate longitudinal changes in downstream concentrations and/or isotopic compositions of multiple chemical contaminants. In addition, we also sampled shallow groundwater at Scotts Level Branch along the flowpath from uplands to the stream channel using transects of piezometers. Methods of sampling for groundwater at Scotts Level Branch and details on groundwater well installation are in (Wood et al., 2022). In each watershed, LSS monitoring was completed within 24 h or on consecutive days with similar streamflow conditions. We did not always move downstream along a watershed during the day; in some cases, we moved upstream or sampled the entire watershed flowpath by multiple teams simultaneously throughout the day (*e.g.*, sampling timing was sometimes different for flowpaths and data collection could sometimes rotate through upstream, downstream, and simultaneous collection along flowpaths). Thus, the chemical patterns along flowpaths were not due to diurnal patterns throughout the day. Our sampling resolution between sampling points varied based on the size of the watershed, but was typically hundreds of meters or a few kilometers; thus, we sampled in detail along the length of each watershed to evaluate where urban degradation occurred and/or restoration activities were implemented. In some cases, these flowpaths extended from headwaters to the stream outflow to receiving waters. Sampling locations for the synoptic sites along each mainstem were chosen based on accessibility, presence of tributary junctions, and positioning of conservation and restoration features.

Characteristics (*i.e.*, stream, sampling dates, longitudinal distance sampled, mainstem and tributary sampling points, and USGS station location and discharge) of each synoptic event are included in Table 2. Water samples were collected at the tributary outflows and at least 100 m downstream from the tributary confluence along the mainstem to ensure well-mixed conditions (almost all data presented in this paper is from along the mainstem). Latitude and longitude for synoptic sites were recorded using GPS systems or applications. Along the Gwynns Falls, our synoptic sampling also included discharge measurements using a Marsh McBirney 2000 (Hach Co., Loveland, CO, United States) velocity meter. LSS monitoring was conducted mostly during baseflow conditions, and there was also targeted sampling after snow events along Rock Creek, Bull Run, and Anacostia River, Campus Creek.

### 2.4 Chemical analyses of streamwater and groundwater samples along the UWC

Major element (*e.g.*, Na, Ca, K, Mg) and trace element (*e.g.*, Mn, Zn, Sr, Cu) concentrations were measured by inductively coupled plasma optical emission spectrometry in an acidified (0.5% highpurity nitric acid) analytical matrix on a Shimadzu Elemental Spectrometer (ICPE-9800). For major element measurements, the acidified samples were nebulized in radial mode (across a plasma flame). For trace element measurements, the acidified samples were nebulized in axial mode (down plasma flame). The instrument was calibrated to the range of trace metals that are commonly observed in urban streams in accordance with analytical guidelines for surface water analysis (Galella et al., 2021). There were no non-detects in the measurements of trace elements we report for these polluted urban streams. Major and trace elements in Gwynns Falls acidified stream samples were analyzed by ICP-MS at the United States Environmental Protection Agency Lab in Ada, Oklahoma, United States. Dissolved organic carbon (DOC), dissolved inorganic carbon (DIC) and total dissolved nitrogen (TDN) were measured using a Shimadzu Total Organic Carbon Analyzer (TOCV CPH/CPN) and total nitrogen module, TNM-1 (Haq et al., 2018). Isotope ratios of ^15^N/^14^N in nitrate in stream samples from the Anacostia River and its tributaries were measured at the UC Davis Stable Isotope Facility. Isotope ratios of ^15^N/14N were measured using a ThermoFinnigan + PreCon trace gas concentration system interfaced to a ThermoScientific Delta V Plus isotope-ratio mass spectrometer.

#### 2.4.1 Dissolved gas concentration measurements along the UWC

Longitudinal synoptic samples for analyses of dissolved CO_2_, CH_4_ and N_2_O were collected during October 2013 using a headspace equilibration protocol. Gas sample collection, laboratory analysis and data processing methods are nearly identical to those described in (Smith et al., 2017). Briefly, at each site we collected a single 115 ml water sample into an airtight syringe, equilibrating dissolved gasses by adding 25 ml of added Ultra High Purity He in the field through a 3-way luer lock valve and shaking for 5 min in order to reach equilibrium. We then immediately injected 20 ml of the headspace into a pre-evacuated Exetainer vial (LabCo Inc Exeter UK) and stored at room temperature until analysis within 1 month of sampling. Sampling included 10% field duplication of synoptic samples, He field blanks, and 5 laboratory gas standards and blanks meant to assess leakage from exetainers during transport. Headspace gas concentrations were analyzed by the United States Environmental Protection Agency Lab in Cincinnati, OH, United States. Concentrations of CO_2_, CH_4_, and N_2_O were measured using a Bruker 450 (Bruker; Billerica, MA, United States) gas chromatograph equipped with a methanizer, flame ionization detector, and electron capture detector (Smith et al., 2017). Instrument detection limits were 100 ppb for N_2_O, 10 ppm for CO_2_, and 0.1 ppm for CH_4_.

#### 2.4.2 Dissolved organic matter characterization using optical properties along the UWC

Excitation-emission and UV spectra were measured on water samples collected from Bull Run using a FluoroMax-4 Spectrofluorometer (Horiba Jobin Yvon, Inc.) and a UV-1800 UV Spectrophotometer (Shimadzu). Excitation–emission matrices (EEMs) were used to calculate indices for DOM sources (terrestrial vs aquatic) and lability (protein vs. humic), including: (1) the humification index (HIX), defined as the ratio of emission intensities in the 435–480 nm and 300–345 nm regions of the EEM for an excitation wavelength of 254 nm (Zsolnay et al., 1999; Ohno, 2002). (2) The biological autochthonous inputs index (BIX), defined as the ratio of emission intensities at 380 nm and 430 nm for an excitation wavelength of 310 nm (Huguet et al., 2009), (3) the fluorescence index (FI), used to track autochthonous versus allochthonous DOM inputs in freshwater environments and defined as the ratio of emission intensities at 470 and 520 nm for an excitation wavelength of 370 nm (McKnight et al., 2001; Cory and McKnight, 2005), and (4) Coble’s Peaks (Coble et al., 2014), including the A peak (excitation/emission ratio of 260 nm/380–460 nm) which is a marker for terrestrial humic-like DOM, the C peak (excitation/ emission ratio of 320–360 nm/420–460 nm) which is a marker for terrestrial fulvic-like DOM, the B and T peaks (excitation/emission ratios of 270–280 nm/320–350 and 330–320 nm, respectively), both indicative of protein-like microbial sources, and the M peak (excitation/emission spectra of 290–310 nm/370–410 nm) a marker for marine-humic like microbial sources (Coble et al., 2014). All indices and peaks were calculated from measured EEMs using R package staRdom (Pucher et al., 2019), accounting for instrument-specific solution interference corrections, as described in (Pucher et al., 2019).

### 2.5 Statistical analyses

Summary statistics were calculated for all chemical constituents measured on each stream. We used linear regression analysis to test for increasing or decreasing trends between chemical concentrations and distance downstream. If concentration versus distance did not follow a linear trend, the polynomial fits in Excel or the curve fitting toolbox in MATLAB (cftool Mathworks, 2001) was used for quantitative solutions for longitudinal patterns in chemical concentrations.

Principal component analysis was used to assess the temporal and spatial chemical cocktails along the flowpath in Bull Run and Gwynns Falls. Before performing PCA, the data was evaluated using histograms and quantile-quantile plots to determine the normality. The data was determined to be positively skewed and log-transformed to avoid violating the normality constraints of PCA (Davis and Sampson, 1986). The data was standardized to have a mean of zero and a standard deviation of one. The PCA was performed in R studio version 1.4 and used the packages FactoMineR and factoextra (Lê et al., 2008). For both analyses, a resampling-based stopping rule (Peres-Neto et al., 2005; Rippy et al., 2017) was used to identify principal components that explained significantly more data variance than expected due to chance (significance determined at a 95% confidence level). Only these significant components were retained and interpreted.

## 3 Results

Results from LSS monitoring showed distinct typologies of different chemical concentrations with distance downstream, and groups of elements with similar patterns showed the potential to form chemical cocktails. The grouping of elements into chemical cocktails can provide useful information on chemical sources and transformations along the flowpath (Kaushal et al., 2018; Kaushal et al., 2020; Kaushal et al., 2022). If all elements show declining longitudinal patterns in the same manner, that could indicate downstream dilution as suggested by the typology (Figure 2). If some elements increase with distance downstream that suggests chemical supply is greater than demand, which is represented in the typology (Figure 2). For example, elements such as B found in detergents can increase due to sewage leaks in urban streams (Kaushal et al., 2011; Kaushal et al., 2020). Ca, Mg and Na can increase further downstream due to impervious surfaces and road salts (represented by increasing trends in the typology). In contrast, elements which originate primarily from natural weathering such as Ba in some cases can decrease downstream (represented by dilution trends in the typology). Redox sensitive elements such as Fe and Mn can increase in dissolved form in the presence of restoration and conservation features such as floodplains and wetlands, which promote denitrification (suggesting transformations and transition zones represented in the typology). Below, we discuss examples of longitudinal synoptic patterns in chemical cocktails along the 10 watershed flowpaths. These longitudinal patterns provide regional examples of longitudinal water quality trends in streams and support the need for developing a typology framework to analyze diverse watershed responses. Regional results from longitudinal studies in streams have implications for monitoring recovery and degradation of urban water quality.

### 3.1 Trends, transitions, and typologies in chemical concentrations along the flowpaths

**FIGURE 4:**
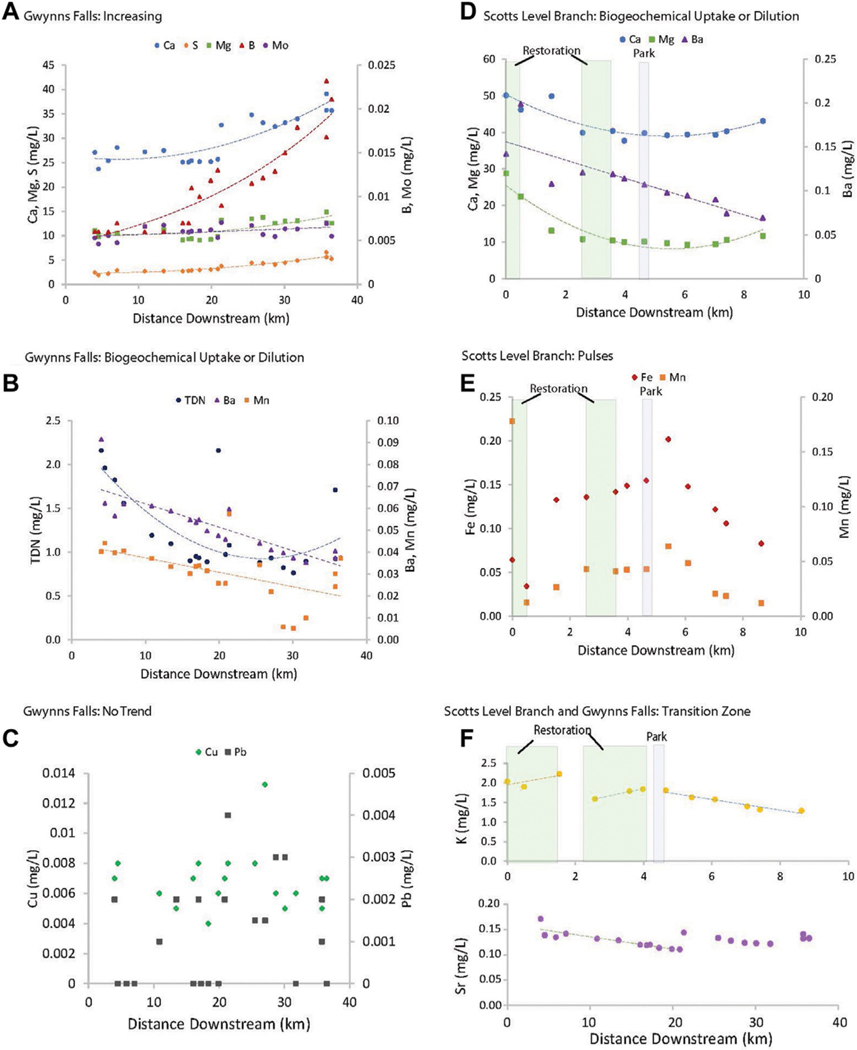
Examples of typologies from longitudinal stream synoptic patterns of chemical cocktailswith increasing distance downstream. Elements with similar longitudinal patterns represent chemical cocktails. In panels 3d–f, we included restoration reaches (green highlight) and a local park (purple highlight) in our sampling. * dashed trendlines are included when *p* < 0.05. Trend line information including equations, p values, and R^2^ values are in Supplemental Table S2.

LSS monitoring illustrated distinct typologies in chemical concentrations along the Gwynns Falls flowpath and one of its major tributaries, Scotts Level Branch (Figure 4). Along the Gwynns Falls flowpath, we observed statistically significant increasing trends for calcium, magnesium, sulfur, boron, and molybdenum (increasing trends of approximately 0.014, 0.005, 0.003, 0.005, 3 × 10^−5^ mg/L per km, respectively) (p< 0.05) (Figure 4A). We also observed significant decreasing trends in TDN, Ba, and Mn concentrations (decreasing trends of approximately −0.002, −0.001, −0.0007 mg/L per km, respectively) (Figure 4B). Redox sensitive elements like Fe and Mn showed a pulse typology with increasing distance downstream and varied in concentrations suggested by the presence of floodplain restoration sites and parks (Figure 4E). Some elements like K and Sr showed transition typologies characterized by stepwise changes with increasing distance downstream (Figure 4F). Some trace elements like Cu and Pb (at very low concentrations) showed no pattern with distance downstream (Figure 4C). Overall, these results suggest that there can be distinct typologies in longitudinal changes in chemical concentrations along stream flowpaths.

**FIGURE 5:**
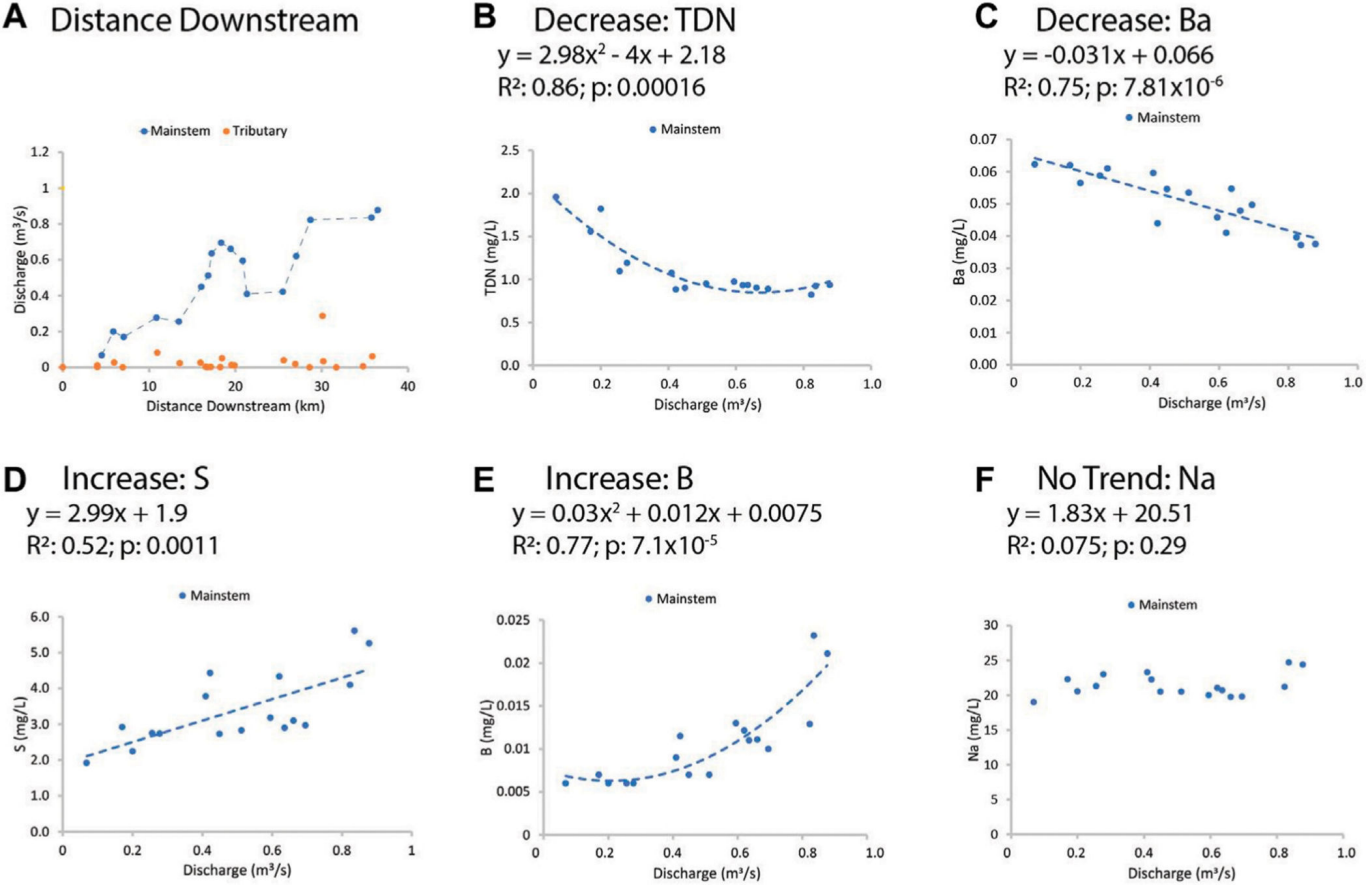
Longitudinal concentration-discharge relationships along synoptic surveys from the Gwynns Falls. Trend line information including equations, p values, and R^2^ values are in Supplemental Table S2.

Streamflow increased longitudinally along the Gwynns Falls, and there were distinct relationships between elemental concentrations and increasing discharge (Figure 5). Not all elements were simply diluted with increasing streamflow along the flowpath (Figure 5). In addition, longitudinal patterns in chemical concentrations along the mainstem did not appear to show abrupt pulses or dilutions from discharge from tributaries (Supplemental Figure S1); a detailed mass balance analysis of loads along the Gwynns Falls mainstem and inputs from tributaries can be found in Kaushal et al., (2014a). Instead, longitudinal concentration-discharge patterns showed distinct patterns and processes along the Gwynns Falls UWC. For example, concentrations of TDN and Ba decreased linearly whereas S concentrations increased linearly and B concentrations increased curvilinearly (Figure 5). There was no evident relationship between streamflow and stable Na concentrations (chemostatic behavior) (Figure 5). Differences in the longitudinal concentration-discharge relationships suggested the possibility to group elemental transport into distinct chemical cocktails along the Gwynns Falls flowpath.

### 3.2 Biogeochemical and geochemical transformations along flowpaths

**FIGURE 6:**
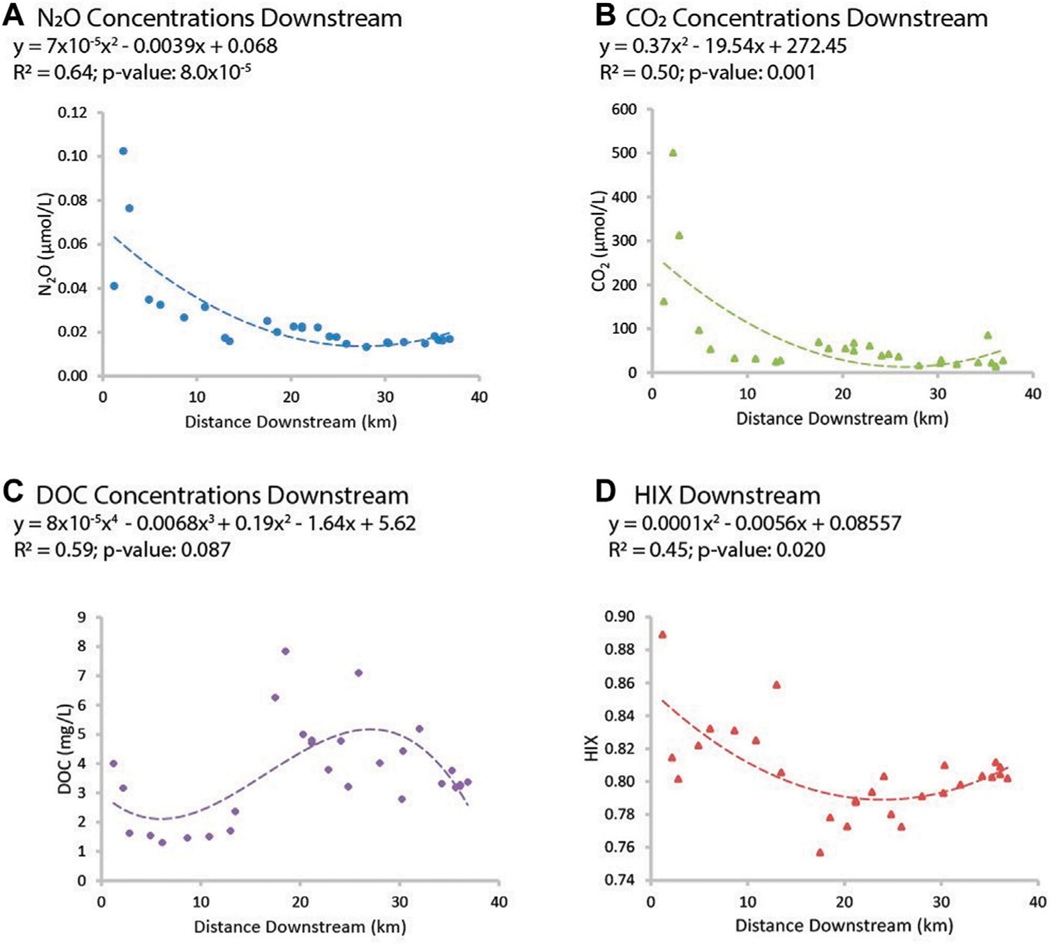
Longitudinal stream synoptic monitoring of biogeochemical transformations and greenhouse gas concentrations within the Gwynns Falls watershed. Urban land use increases with increasing distance downstream. CO_2_, N_2_O, and humification index (HIX) appear to show a downstream dilution typology whereas DOC appears to show a transition zone typology where it increases abruptly downstream. Our objective was to illustrate the typologies using regional data but a t-test could be also used to compare the upstream and downstream DOC data. Trend line information including equations, p values, and R^2^ values are in Supplemental Table S2.

LSS monitoring revealed biogeochemical transformations and transition zones along the Gwynns Falls flowpath (Figure 6). Along the Gwynns Falls flowpath, we observed polynomial declining trends in concentrations of CO_2_ and N_2_O with increasing distance downstream (Figure 6). There was also a decline in the humification index of organic matter (HIX), which suggested a shift from terrestrial to aquatic sources of DOC along the flowpath (Smith and Kaushal, 2015) (Figure 6). In addition, there was an abrupt transition typology and increase in DOC concentrations approximately 20 km along the Gwynns Falls flowpath (Figure 6). Thus, CO_2_, N_2_O, and HIX appeared to show a downstream dilution typology (Figure 2) whereas DOC appeared to show a transition zone typology (Figure 2) where it increased abruptly downstream.

**FIGURE 7:**
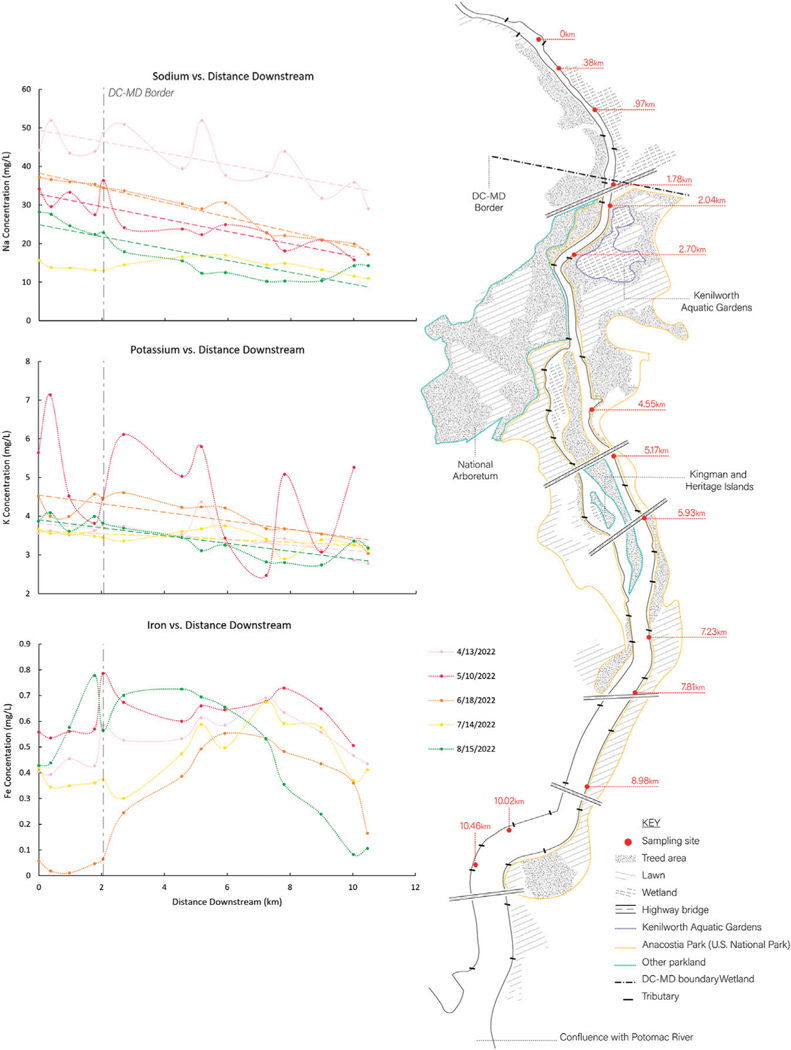
Longitudinal stream synoptic monitoring of Na, K, and Fe along the Anacostia watershed continuum across different seasons. There was a decreasing/dilution typology for Na and K, as the river flowed through conserved forests and wetlands in Anacostia National Park. However, there was a pulse typology in dissolved Fe, as the river flowed through conservation areas likely due to wetlands and changes in redox conditions. Statistically significant relationships are indicated by lines. Trend line information including equations, p values, and R^2^ values are in Supplemental Table S2.

LSS monitoring revealed biogeochemical transformations along the Anacostia River flowpath (Figure 7). The importance of biogeochemical transformations along the Anacostia River flowpath can be explored using LSS monitoring across time. Na and K concentrations typically showed significant decreases longitudinally downstream (Figure 7), as the river flowed through conservation areas of Anacostia National Park with tidal wetlands and forests. Concentrations of K, a biologically limiting nutrient, were substantially lower than Na along the flowpath (Figure 7). In contrast, concentrations of dissolved Fe showed a pulse typology as the river flowed through adjacent forest and wetland areas likely due to changes in redox conditions and biogeochemical reactions (similar to pulses in Fe and Mn in restoration features at Scotts Level Branch) (Figure 7).

**FIGURE 8:**
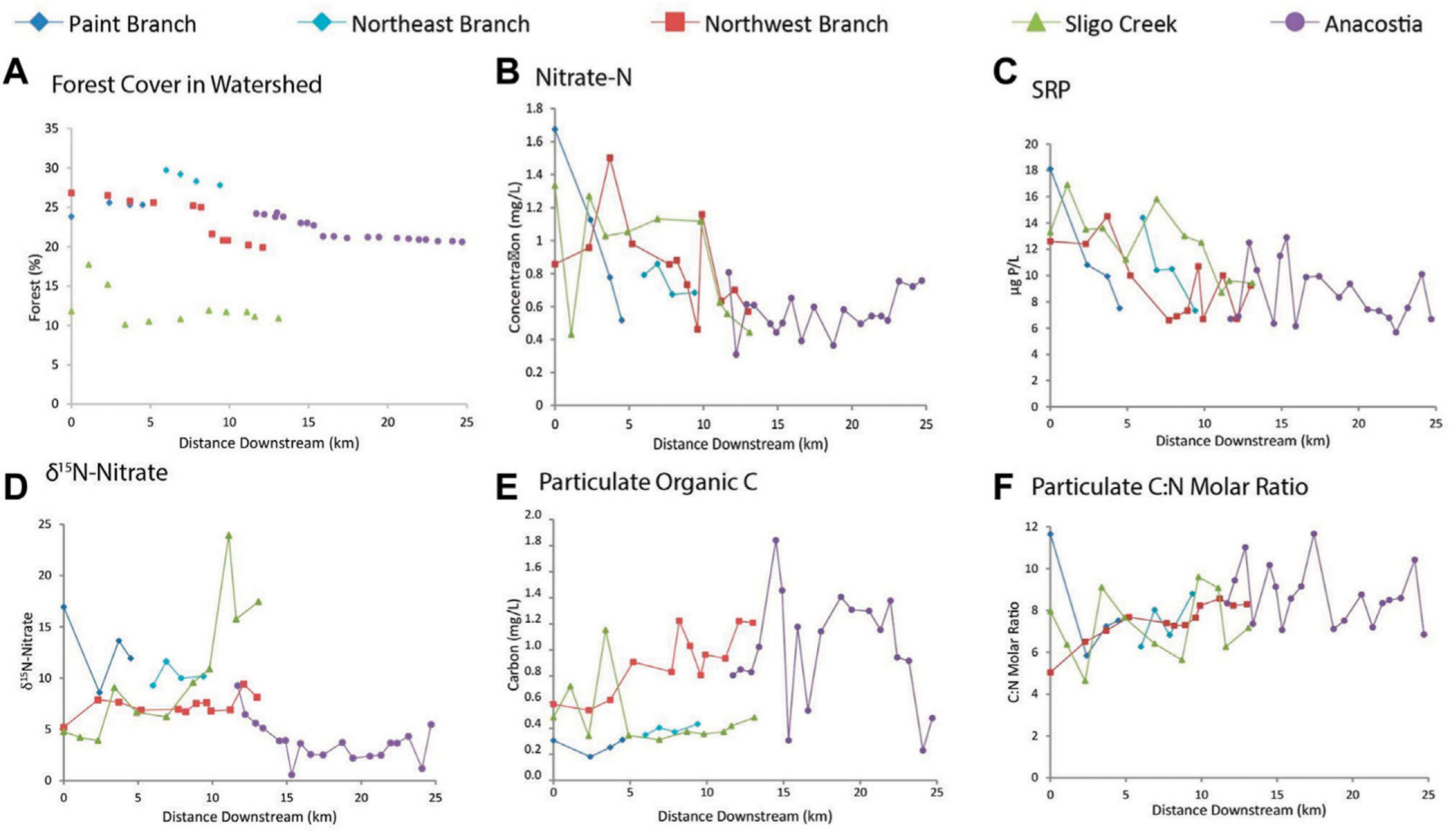
Longitudinal stream synoptic monitoring reveals biogeochemical patterns and typologies along the Anacostia watershed continuum. Sligo Creek is a subwatershed of the Northwest Branch Anacostia. Paint Branch is a subwatershed of the Northeast Branch Anacostia.

LSS monitoring suggested potential transformations of nutrients and organic matter along the Anacostia River flowpath (similar to changes in dissolved Fe as discussed above), as water flowed from degraded suburban and urban headwaters into conserved tidal wetlands and forests (Figure 8). Farther downstream (particularly along the Northwest Branch through Burnt Mills Park and along the Anacostia mainstem through Anacostia National Park), there appeared to be uptake of nutrients and increases in organic matter concentrations (Figure 8). Nitrate (NO_3_^−^) and soluble reactive phosphorus (SRP) declined rapidly along the Anacostia mainstem, while particulate organic carbon and the C:N ratio of particulate organic matter increased (which suggests the importance of biological uptake of nutrients and transformation into organic matter) (Figure 8). Paint Branch and Sligo Creek showed significantly elevated δ^15^N-NO_3_^−^ above +10 within the range of sewage or caused by fractionation (Kaushal et al., 2011) (Figure 8). The δ^15^N-NO_3_^−^ (Figure 8D) decreased along flowpaths draining extensive riparian conservation areas such as the Northwest Branch (through Burnt Mills Park) and Anacostia mainstem through Anacostia National Park. Conversely, the δ^15^N-NO_3_ increased along urban flowpaths with minimal riparian buffers such as Sligo Creek, the Northeast Branch, and Paint Branch (Figure 8D).

**FIGURE 9:**
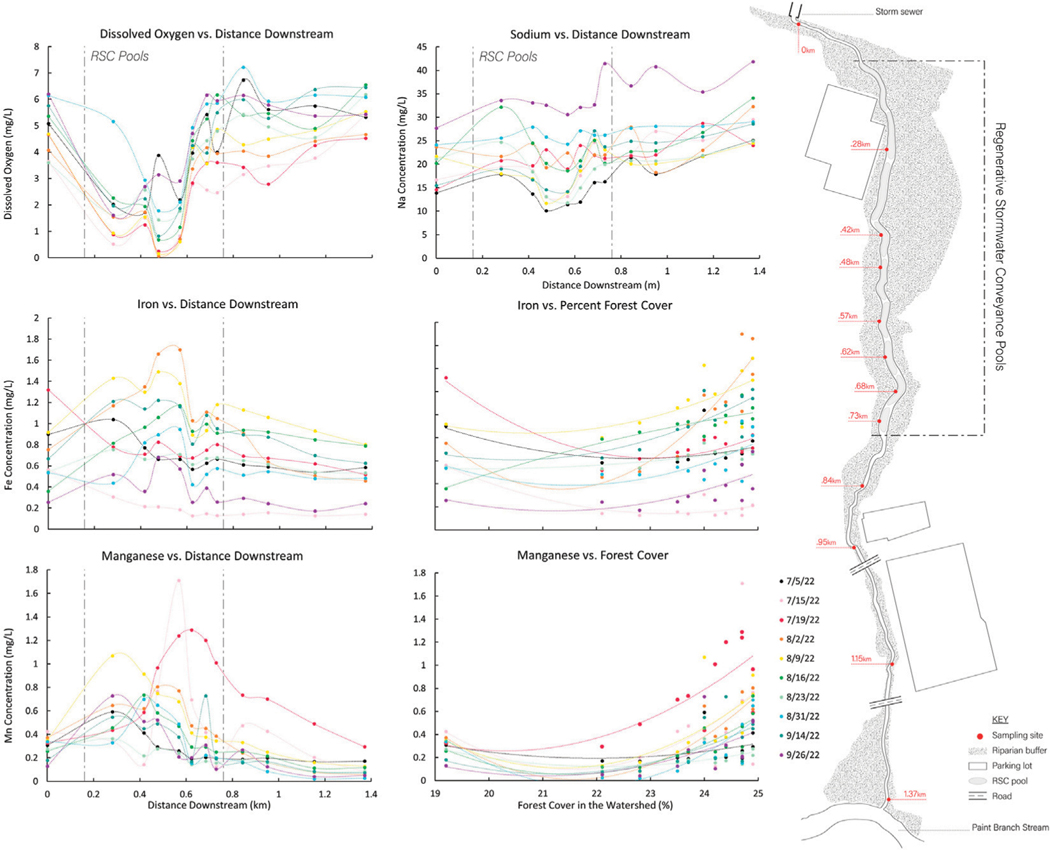
Longitudinal stream synoptic patterns of dissolved oxygen, Fe, Mn, and Na along the Campus Creek watershed continuum. Across all seasons, there were declines in dissolved oxygen as water flowed through restored stream reaches that were hydrologically connected to floodplains. There were increasing concentrations of dissolved Fe and Mn in restored stream reaches due to decreased oxygen levels and reducing conditions. Na concentrations decreased and were attenuated or diluted along stream reaches that were hydrologically connected to floodplains, but then increased downstream of the restoration. There were relationships between Fe and Mn and % forest cover; trend line information including equations and R^2^ values are in Supplemental Table S2.

LSS monitoring was also able to delineate the spatial and temporal extent of biogeochemical transformations and transition zones associated with stream and floodplain restoration activities along the Campus Creek flowpath (Figure 9). For example, there were substantial declines in dissolved oxygen along the Campus Creek flowpath, as it flowed through reaches with regenerative stormwater conveyance (pools and wetlands with longer hydrologic residence times) (Figure 9). During summer months, dissolved oxygen concentrations were less than 2 mg/L indicating hypoxia (Figure 9). As dissolved oxygen declined along the regenerative stormwater conveyance pools and hydrologically connected floodplains, there were increases in concentrations of dissolved Fe and Mn (similar to restored reaches in Scotts Level Branch and forest and wetland conservation areas in Anacostia National Park) (Figure 9). This was likely due to low oxygen conditions and reduction of Fe and Mn to soluble forms. There was also a relationship between % forest and shrub cover in the watershed and concentrations of Fe and Mn in Campus Creek (Figure 9). In contrast to redox sensitive elements, Na showed a dilution typology as the stream flowed through the regenerative stormwater conveyance pools, which may be expected from biologically conservative elements.

### 3.3 Attenuation of wastewater, road salt, and urban stormwater along flowpaths

**FIGURE 10:**
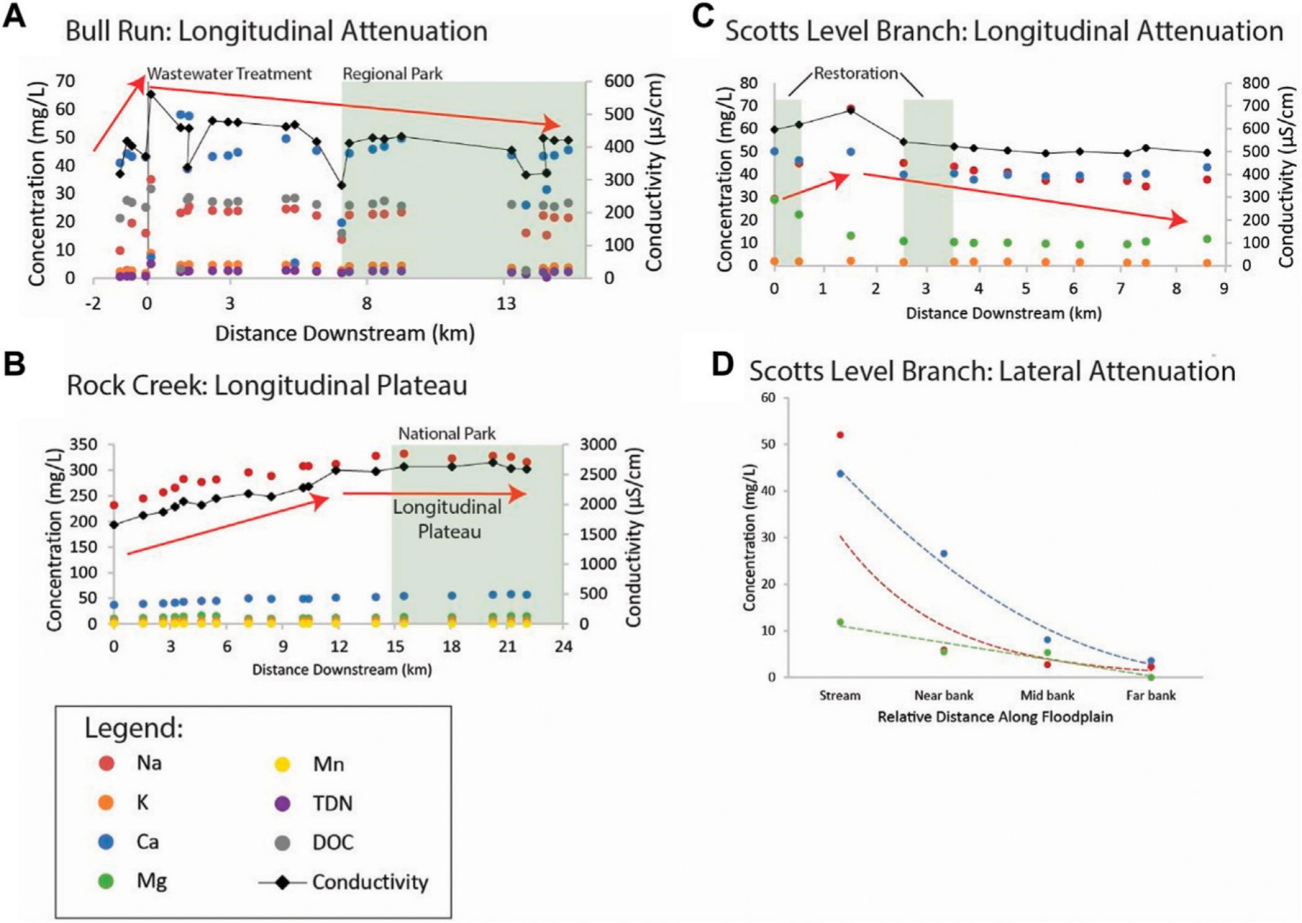
Longitudinal stream synoptic monitoring can trace the attenuation of chemical cocktails along longitudinal and lateral flowpaths. Chemical cocktails can show decreasing or plateau typologies along flowpaths that are influenced by wastewater effluent discharge [Panel (**A**), indicated by the vertical line at 0 m distance downstream], road salt events [Panel (**B**)], and restoration along longitudinal and lateral flowpaths [Panel (**C,D**)]. Trend line information including equations, p values, and R^2^ values are in Supplemental Table S2.

**FIGURE 11:**
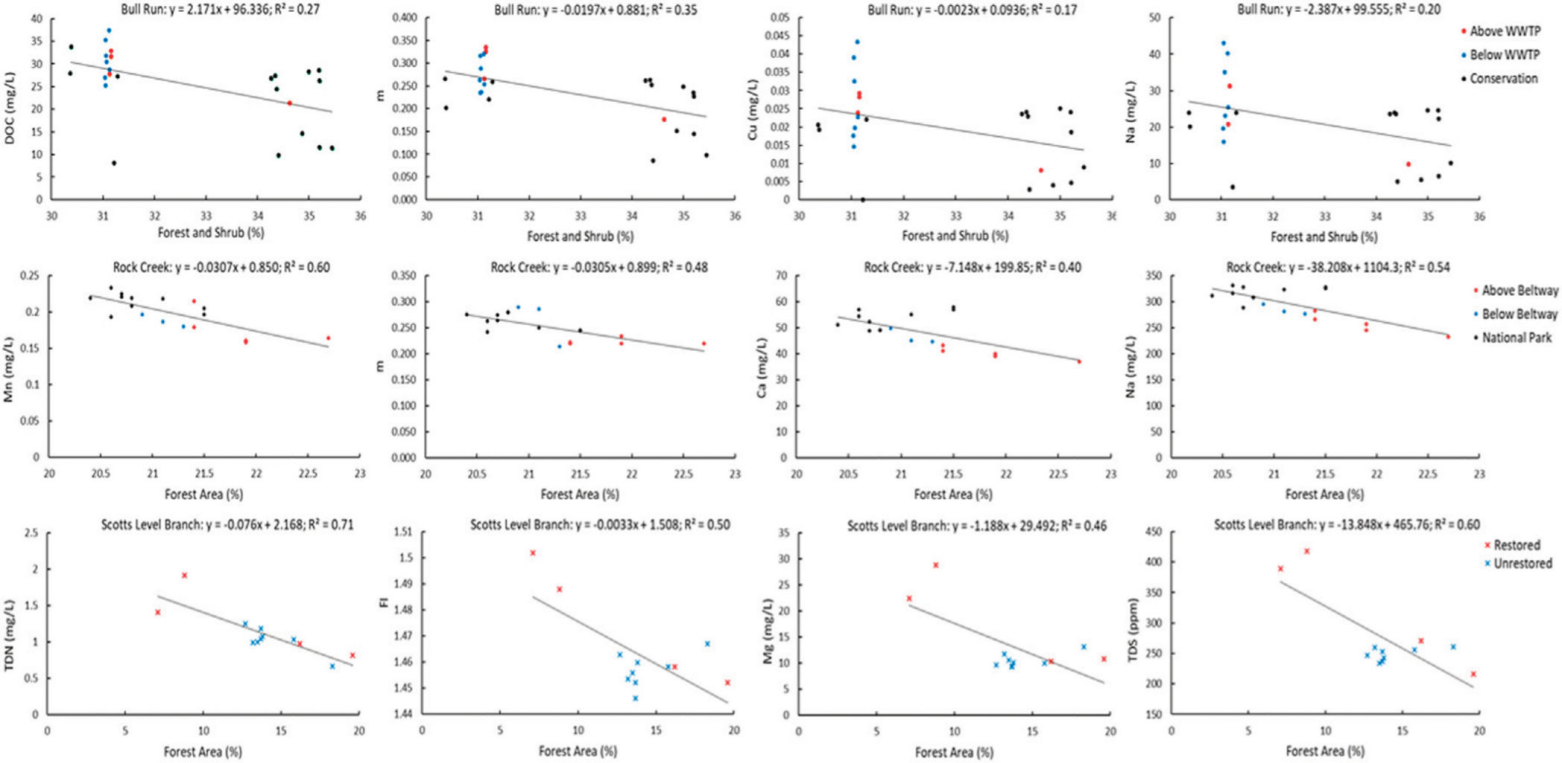
Examples of statistical relationships between chemical concentrations and % forest and shrub cover in the Bull Run, Rock Creek, and Scotts Level watersheds. FI is an abbreviation for fluorescence index and m represents a peak in dissolved organic matter fluorescence. The indices of organic matter composition are described in the methods section.

Results from LSS monitoring also revealed attenuation of pollution from wastewater, road salt, and urban stormwater along flowpaths through restoration and conservation areas (Figure 10). There were also significant linear decreasing relationships between % watershed forest and shrub cover and chemical concentrations in these same watersheds (Figure 11). We observed two types of rapid declining patterns in concentrations of many chemical constituents (Na, K, Ca, Mg, Mn, TDN, DOC, conductivity) along the Bull Run flowpath downstream of the UOSA wastewater treatment plant. First, there was a very rapid initial decline in concentrations between the UOSA effluent and our first sampling point downstream likely due to dilution as a physical driver, as evidenced by the consistent and similar decreasing slopes for chemicals between UOSA at this first sampling station (Figure 10A). Different chemicals showed varying rates of decline and transport lengths likely due to their reactivity, changes in their phases (dissolved vs. particulate), and other biogeochemical processes, as Bull Run flowed through extensive forest and wetland areas in a regional park. Please see Figure 11 for examples of significant relationships between chemical concentrations and % forest and shrub cover in Bull Run.

A plateau typology in chemical concentrations was observed in Rock Creek along a stream flowpath through conservation areas and a national park. During a winter road salt event, there was increasing specific conductance and concentrations of major and trace elements such as Na, Ca and Mn with distance downstream (Figure 10B). However, these chemical concentrations appeared to plateau and remain more constant once Rock Creek flowed through the heavily forested National Park with extensive forest riparian buffers (Figure 10B). Please see Figure 11 for examples of significant relationships between chemical concentrations and % forest and shrub cover in Rock Creek.

Finally, we observed a decreasing typology of urban stormwater pollution in Scotts Level Branch for a portion of the flowpath, where there is a stream restoration project involving hydrologic reconnection of the floodplain with the stream. After a rainstorm, we observed declines in specific conductance and concentrations of carbon, nutrients, and major elements in Scotts Level Branch, as it flowed from an urban storm drain through restored floodplains and a local park; however, there were too few sampling points along this particular stream reach to analyze rates of attenuation (Figure 10C). Please see Figure 11 for examples of significant relationships between chemical concentrations and % forest and shrub cover in Scotts Level Branch. There was a coinciding increase in Fe and Mn concentrations downstream of the restored stream-floodplain complexes as mentioned previously (Figure 4), likely due to a change in redox conditions favoring Fe and Mn dissolution and denitrification. Along lateral groundwater flowpaths, there was also a decline in Na with increasing distance away from the stream along the riparian zone in Scotts Level Branch (Figure 10D). Attenuation of chemical cocktails along the Scotts Level Branch flowpath are likely due to increased biogeochemical transformations such as denitrification and cation exchange in floodplain soils.

### 3.4 Tracking transport of multiple chemical cocktails along flowpaths

**FIGURE 12:**
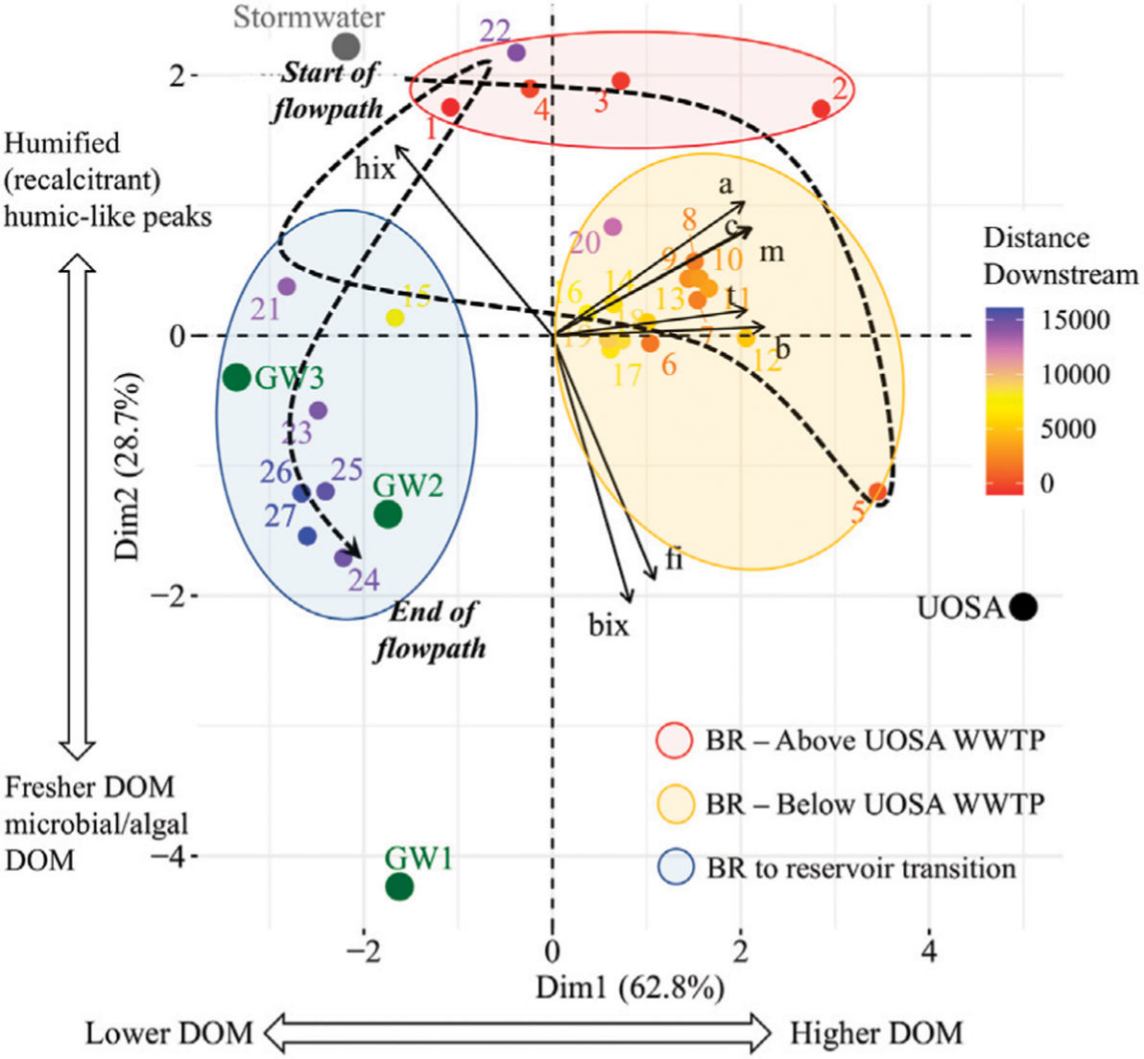
Longitudinal stream synoptic monitoring traces the attenuation of organic chemical cocktails from wastewater. PCA shows the influence of the wastewater treatment effluent. The dots indicate samples that were collected along the mainstem of the flowpath, with the dot labeled 1 at the start of the flowpath and increasing with distance downstream. The samples in green labeled “GW” indicate samples that were collected from groundwater seeps. The dashed line connects the points from sample 1 to the end of the flowpath. The ellipses in the figures encapsulate statistically different regions along the flowpath identified using hierarchical clustering of principal components. They correspond to changes in the dominant land use type along the flowpath and illustrate how the dominant chemical cocktail changes with different land uses. With increasing distance downstream, the organic matter changes based on the influences of the watershed (*i.e.*, UOSAwastewater treatment plant, forested reach). At the start of the flowpath, dissolved organic matter had a stormwater signature and then was pulled toward a wastewater effluent signature and then finished with a forested groundwater signature in Bull Run Regional Park.

**FIGURE 13:**
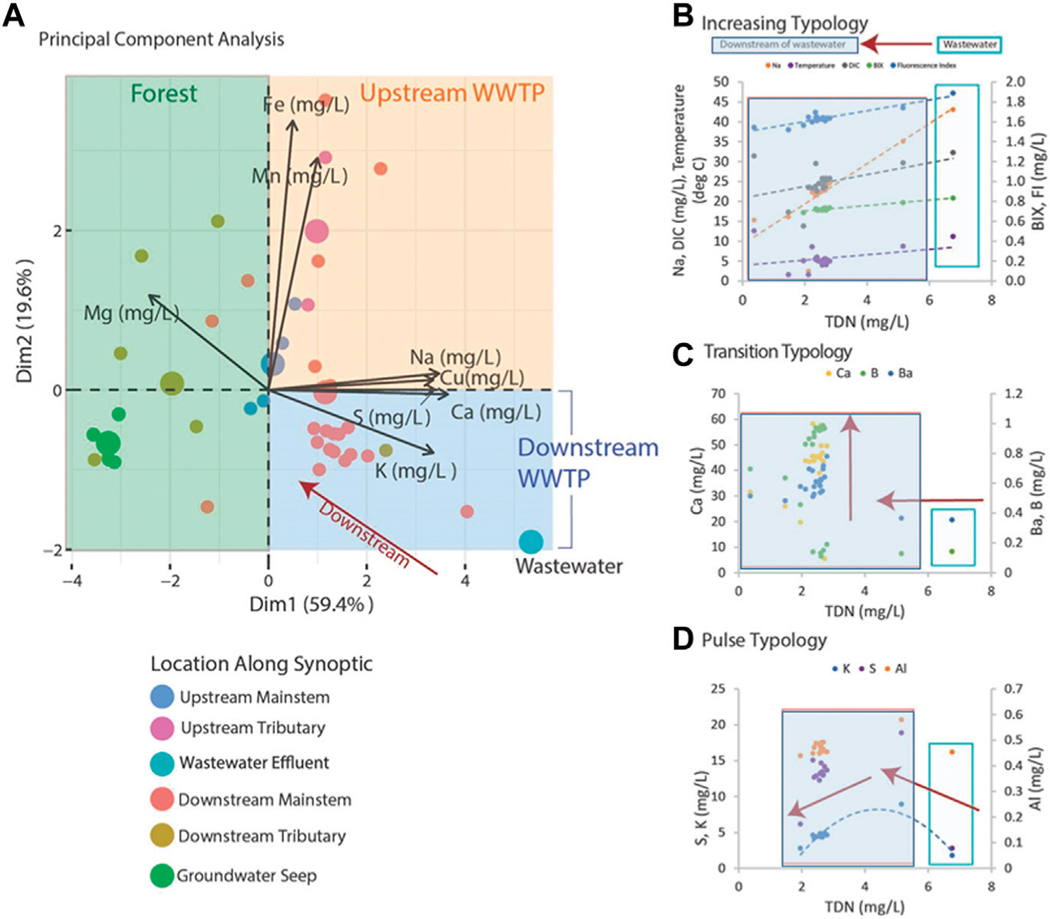
Longitudinal stream synoptic monitoring can trace the attenuation of inorganic, ionic chemical cocktails. Wastewater effluent has a distinct chemical cocktail, which influences the downstream chemical cocktails [Panel **(A)**. Panels (**B–D**)] show trends from the wastewater effluent signal with distance downstream. Different variable patterns appear from the wastewater effluent with the high TDN downstream. The color coded regions correspond to general quadrants of PC space that have been colored to reflect where the majority of samples were collected (*i.e.*, they are approximately guided by the three regions from Figure 12). Trend line information including equations, p values, and R^2^ values are in Supplemental Table S2.

Applications of LSS monitoring showed potential to track fate and transport of multiple chemical cocktails along flowpaths using typologies (similar longitudinal patterns among chemicals). Principal component analysis also revealed that there were distinct chemical cocktails of organic matter and elemental mixtures formed along the Bull Run flowpath (Figures 12, 13). In Figure 12, the dashed line indicates movement along the flowpath from upstream to downstream. The ellipses represent the land use changes along the flowpath. The different regions they encapsulate were identified statistically using hierarchical clustering of principal components (R, function HCPC). They were subsequently characterized (interpreted) both visually using maps along the flowpath and analytically by measuring the changes in impervious surface cover, forest cover, and riparian buffer width in the region. Dissolved organic matter downstream of the UOSA wastewater treatment plant was enriched in the biological freshness index (BIX) and protein-rich peaks B and T, which can indicate more labile DOM from microbial sources (Huguet et al., 2009; Coble et al., 2014). In contrast, sites with DOM showing higher humification index (HIX), which can indicate more refractory DOM from humic substances (Zsolnay et al., 1999; Ohno, 2002), were located either upstream of UOSA or considerably further downstream near the reservoir in Bull Run Regional Park (Figure 12). An alternative interpretation is that B and T peaks were correlated with PC1, but A, C, and M peaks have very similar loading strengths. This may suggest that all peaks were enriched, which can be explained as higher concentrations, which result in higher intensities for all peaks. Indices that drive PC2, or ratios of peaks can give concentration-independent metrics. At the start of the flowpath, dissolved organic matter had a stormwater signature that changed to a wastewater effluent signature, and then finished with a forested groundwater signature in Bull Run Regional Park. Thus, there were distinct changes in chemical cocktails of organic matter that could be traced along the Bull Run flowpath using LSS monitoring (see black trace in Figure 12).

LSS monitoring was also able to track transitions in chemical cocktails upstream of the wastewater treatment plant, immediately downstream of the UOSA wastewater treatment plant, and within forest and wetland conservation areas in the regional park (Figure 13). Elemental mixtures immediately downstream of UOSA were enriched in DOC, Na, TDN, Cu compared with elemental mixtures at sites within the forests and wetlands of Bull Run Regional Park (Figure 13). Given that N is a limiting nutrient in ecosystems, N from wastewater treatment plant discharges can also serve as a reactive tracer in downstream ecosystems (Figure 13). There were significant positive linear relationships between TDN concentrations and a variety of chemical parameters (DOC, Na, humic-rich peaks A and C in organic matter, and Cu) along Bull Run (Figure 13). There were other typologies of asymptotic trends and negative polynomial trends for chemical cocktails downstream (Figure 13). Results from longitudinal patterns illustrated transformation of different chemical cocktails with transitions spanning from urban wastewater effluent to downstream forest/wetland conditions.

## 4 Discussion

Our paper presented a collection of regional case-studies on streamwater chemistry trends along watershed flowpaths and in combination with the literature demonstrates and illustrates a typology of common longitudinal water quality patterns (Figure 2; Table 1). Our typology of longitudinal water quality patterns has significant value for helping inform appropriate stream monitoring regimes and comparing longitudinal patterns across streams. Our results highlight the importance of applying longitudinal stream synoptic (LSS) monitoring to understand transport and transformation of chemical cocktails along flowpaths. A growing number of studies demonstrates the value of developing LSS monitoring applications for evaluating water quality (*e.g.*, Gove et al., 2001, Wayland et al., 2003, Marti et al., 2004, Kaushal et al., 2014a, Newcomer Johnson et al., 2014, Ledford and Lautz, 2015; Pennino et al., 2016a; Pennino et al., 2016b; Gabor et al., 2017, Leng et al., 2021, Hintz et al., 2022a). Although less understood, these longitudinal synoptic patterns can be represented by empirical relationships (linear and curvilinear trends, piecewise trends, stepwise changes, and others), which provide further ability to quantitatively predict trends and transitions in water quality along stream and river segments. More work is necessary to investigate applications of longitudinal synoptic monitoring in water quality studies and statistical approaches for analyzing longitudinal water quality data sets along different stream segments and potential linkages between surrounding land use and local management and restoration interventions.

In particular, our results suggest that longitudinal synoptic patterns of multiple chemicals can be particularly useful in investigating effects of watershed restoration and management activities along stream flowpaths. For example, downstream degradation or restoration in water quality along flowpaths can be detected using a variety of approaches involving chemical cocktails: (1) longitudinal differences in chemical cocktails, (2) relationships between chemical cocktails with distance downstream and upstream watershed land use (*e.g.*, % forest cover, presence of parks and conservation areas, *etc.*), (3) inverse relationships between chemical cocktails representing organic substrates and biogeochemical reactants (organic matter and nutrients), and (4) applications of principal components analysis to delineate and track chemical cocktails along flowpaths. Below, we explore longitudinal patterns of chemical cocktails along flowpaths and potential underlying processes and applications.

### 4.1 Towards developing a typology for analyzing chemical patterns along stream flowpaths

We observed longitudinal patterns in chemical concentrations along the urban watershed continuum (UWC), which can be classified into increasing, decreasing, transitions, or no trends (Figure 2; Table 1). The typologies of longitudinal patterns can be influenced by a variety of factors depending on land use, geology, reactivity of chemical constituents, hydrology, and time (Table 1). Increasing longitudinal trends can occur downstream when biogeochemical supply exceeds the attenuation capacity of stream ecosystems (biological uptake, dilution, ion exchange) (*e.g.*, Gove et al., 2001; Bhatt and McDowell, 2007; Kaushal et al., 2017) (Table 1). Decreasing longitudinal trends can be due to dilution, biological uptake, or ion exchange along flowpaths (*e.g.*, Marti et al., 2004; Welty et al., 2023) (Table 1). There can be abrupt longitudinal transitions in water quality caused by shifts in surrounding land use, management, hydrology, underlying geology, etc. (Table 1). There can also be idiosyncratic, erratic, and pulsed inputs (e.g. Sivirichi et al., 2011; Pennino et al., 2016a; Pennino et al., 2016b) (Table 1). The absence of longitudinal trends can also be due to stable steady state conditions (importance of groundwater contributions). Chemostatic behavior has been used to refer to steady state chemical behavior over time; cases where ion concentrations, for example, do not change with time, even after a major intervention (presumably due to very diverse flow paths and transit times). However, it is important to note that chemostatic behavior can also be used to describe spatial patterns along flowpaths (Hale and Godsey, 2019). We discuss some examples of longitudinal patterns and processes below:

1. *Increasing trends in concentrations along flowpaths*–We observed increasing downstream trends in some elemental concentrations along the Gwynns Falls (Ca, S, Mg, B, Mo) (Figure 4). These elements could accumulate in groundwater and lead to increasing downstream trends (Ledford and Lautz, 2015; Gabor et al., 2017) or there could be increasing contributions to surface waters from downstream sewage leaks, weathering of impervious surfaces, *etc*. Base cation and chloride concentrations can increase longitudinally along streams due to increased inputs from groundwater (Gabor et al., 2017), increased road salt accumulation in floodplains (Ledford and Lautz, 2015), increased ion exchange (Kaushal et al., 2022), and weathering from impervious surfaces (Kaushal et al., 2017). (Figures 1, 2). Organic carbon concentrations may also increase along some stream flowpaths due to increased terrestrial inputs (*e.g.*, Sabater et al., 1993) or increased production of algal and bacteria (Finlay, 2011; Kaushal et al., 2014a).
2. *Decreasing trends in concentrations along flowpaths*–We observed decreasing TDN trends along the Gwynns Falls and Bull Run (Figure 4), and decreasing nitrate and soluble reactive P trends along the Anacostia (Figure 8), which were all likely due to biological uptake (*e.g.*, Kaushal et al., 2014a, Newcomer Johnson et al., 2014, Ledford and Lautz, 2015; Pennino et al., 2016a; Pennino et al., 2016b). Nitrogen concentrations can decrease along stream flowpaths due to increased biological uptake and/or biological transformations from non-point sources and wastewater sources (*e.g.*, Marti et al., 2004, Haggard et al., 2005, Kaushal et al., 2014a, Ledford and Lautz, 2015; Pennino et al., 2016a; Pennino et al., 2016b) (Figure 2). Organic carbon concentrations can also decrease longitudinally along some rivers due to decreasing downstream wetland cover (Duan et al., 2017). The role of physical dilution as a driver of decreasing trends can also be explored, if the slopes in a conservative tracer or multiple chemical analytes are roughly equal across analytes.
3. *Steady state concentrations (chemostasis) along flowpaths*–We observed that Na concentrations remained relatively stable in the Gwynns Falls with increasing downstream streamflow (chemostatic behavior) (Figure 5). In less disturbed watersheds, organic carbon concentrations can show stable chemostatic conditions longitudinally along flowpaths, where constant inputs are equal to attenuation rates (Hale and Godsey, 2019) (Figure 2),
4. *Abruptly transitioning, stepwise, or piecewise trends in concentrations along flowpaths*–We observed spatial transitions in chemical cocktails along flowpaths through restoration and conservation zones. As one example, we observed rapid transitional increases in Fe and Mn concentrations (also may be considered pulse typologies) in Scotts Level Branch and Campus Creek as the stream flowed through restored stream reaches, which were hydrologically connected to floodplains and wetlands (Figures 4, 9). This was likely due to reducing conditions favoring denitrification (Kaushal et al., 2008b; Newcomer Johnson et al., 2014; Newcomer Johnson et al., 2016; McMillan and Noe, 2017) and increased release of soluble forms of Fe and Mn in these stream reaches surrounded by forests and wetlands. Other work has suggested rapid transitions in chemical concentrations along flowpaths spanning meters to kilometers due to changes in land use, management, and biogeochemical hot spots (*e.g.*, Sivirichi et al., 2011; Ledford and Lautz, 2015; Loken et al., 2018; Hintz et al., 2022a) (Figure 2; Table 1).
5. *Peaks and pulses in concentrations along flowpaths*–We observed erratic pulses in concentrations of trace metals along the Gwynns Falls (Figure 4). Wastewater leaks and other pollution sources can cause erratic peaks and pulses in chemical concentrations along flowpaths (Kaushal and Belt, 2012), and there can be longitudinal peaks and pulses of greenhouse gasses along rivers downstream of wastewater treatment plants (Jin et al., 2018) (Figure 2).
6. *Plateaus in concentrations along flowpaths*–We observed plateaus in concentrations of salts and metals along the Rock Creek flowpath through a forested national park following winter road salt events (Figure 10). Concentrations of salts and metals would have likely continued increasing further downstream, as the stream flowed through urban Washington D.C., if there was no national park. There can be saturation of biogeochemical demand for certain chemicals above thresholds leading to saturation and plateaus in chemical concentrations (Haggard et al., 2001; Loken et al., 2018) (Figure 2).

For a more complete list of typologies and classifications of synoptic-scale patterns based on observations from the literature, please see Figure 2; Table 1.

### 4.2 Analyzing biogeochemical patterns and processes along flowpaths across time

Time, seasonality, and specific pollution events are other important factors influencing LSS patterns. LSS monitoring showed relatively consistent longitudinal decreasing typologies in Na and K concentrations along the Anacostia watershed during spring and summer baseflow, as the river flowed through Anacostia National Park (Figure 7). In contrast, concentrations of Fe showed a pulse typology as the Anacostia River flowed through Anacostia National Park, which was likely due to Fe reduction due to extensive fringing wetlands (Figure 7). In a similar manner to the Anacostia River, there were also similar and consistent longitudinal patterns (a pulse typology) in concentrations of dissolved oxygen, Fe, and Mn along the Campus Creek flowpath, as water flowed through hydrologically connected floodplains and regenerative stormwater conveyance pools (Figure 9). In addition to the effects of winter road salt applications, longitudinal patterns may change with hydrologic conditions over time. Our analysis of relationships between longitudinal patterns in streamflow and elemental concentrations showed that downstream concentration-discharge relationships along flowpaths could be specific to certain chemical cocktails (Figure 5); these downstream concentration-discharge relationships are influenced by similar sources, flowpaths, and reactivity. For example, our previous work along the Gwynns Falls flowpath has shown longitudinal declines in concentrations and fluxes of chemical cocktails of metals and ions due to changes in downstream dilution and groundwater recharge patterns (Kaushal et al., 2018). Analyzing changes in longitudinal patterns in water quality within the same watersheds across varying streamflow conditions has the potential to reveal insights into the importance of hydrologic processes influencing longitudinal patterns (*e.g.*, Figure 5 shows diverse longitudinal concentration-discharge relationships for chemicals influenced by attenuation, dilution, flushing, chemostasis from chronic groundwater inputs, and other processes).

### 4.3 LSS monitoring reveals biogeochemical transformations along flowpaths

LSS monitoring has considerable potential to reveal biogeochemical transformations and transition zones along flowpaths. We discuss some examples below.

#### Transformations of carbon and nutrients along flowpaths –

Our results suggested coupled nutrient uptake and carbon accumulation along some urban watershed flowpaths. We observed downstream declining concentrations of nitrogen along flowpaths of the Gwynns Falls, Scotts Level Branch, Anacostia River mainstem, and Bull Run (*e.g.*, Figures 4, 8, 13). There were also longitudinal changes in the quality and composition of organic matter along the Bull Run flowpath (Figure 12). Significant quantities of nitrogen can be retained and transformed *via* algal and microbial uptake and denitrification along watershed flowpaths (*e.g.*, Marti et al., 2004; Haggard et al., 2005; Newcomer Johnson et al., 2014; Kaushal et al., 2014a; Ledford and Lautz, 2015; Jin et al., 2018). Nitrogen uptake along flowpaths is influenced by stream size, temperature, organic matter availability, stream velocity and turbulence, nitrogen concentrations, and other factors (*e.g.*, Dodds et al., 2002; Hall and Tank, 2003; Wollheim et al., 2008; Grant et al., 2018; Loken et al., 2018). Along the Anacostia River watersheds, we observed coinciding increases in dissolved organic carbon and particulate organic carbon and/or increasing C:N ratios along the flowpath as nutrient concentrations declined longitudinally (Figure 8). Similarly, there was a decline in nitrogen concentrations along the Bull Run flowpath (Figure 10) and coinciding changes in organic matter quantity and quality, as revealed by fluorescence spectroscopy (Figure 12). Previous work has observed increases in DOC concentrations with increasing urbanization along the Gwynns Falls (Kaushal and Belt, 2012; Kaushal et al., 2014a) and other work has observed longitudinal increases in organic matter due to the importance of floodplain wetlands and bacterial density (Sabater et al., 1993). Previous work has also shown strong downstream linkages between C and N uptake and spiraling along forested and human-impacted streams (*e.g.*, Bernhardt and Likens, 2002; Brookshire et al., 2005; Kaushal et al., 2014a; Plont et al., 2020).

We also observed increasing downstream trends in organic matter along the Gwynns Falls and Anacostia flowpaths (Figures 6, 8). As urban flowpaths are overloaded with organic matter and nutrients (Mallin et al., 2006; Blaszczak et al., 2019a; Blaszczak et al., 2019b; Carter et al., 2021), the capacity for stream metabolism of carbon and nitrogen can become saturated, thereby contributing to transport of excess organic matter and nutrients further downstream (Marti et al., 2004; Wollheim et al., 2008; Wollheim et al., 2018). Increased nutrient concentrations along human-dominated river continuums cause eutrophication and hypoxia (Diaz and Rosenberg, 2008; Rabalais et al., 2010). However, less work has considered production and accumulation of reactive organic matter along stream and river flowpaths as an additional cause of downstream hypoxia in coastal waters (Mallin et al., 2006; Blaszczak et al., 2019a; Blaszczak et al., 2019b; Carter et al., 2021). Oxidation of bioavailable organic matter can further contribute to biological oxygen demand, increase organic nutrient delivery and primary production, and contribute to harmful algal blooms (Rabalais et al., 2010). Impacts of urbanization on organic matter overloads along flowpaths warrant further research and consideration in water quality (*e.g.*, Mallin et al., 2006, Kaushal and Belt, 2012, Kaushal et al., 2014b; Blaszczak et al., 2019a; Blaszczak et al., 2019b; Carter et al., 2021).

#### Transformations of greenhouse gasses along flowpaths –

We observed longitudinal changes in concentrations in greenhouse gasses along the Gwynns Falls (decreasing and transition typologies) (Figure 6). Streams and rivers can be important sources of GHGs to the atmosphere due microbial processing of terrestrial inputs of organic matter and inorganic nitrogen into globally substantial quantities of CO_2_ and N_2_O to the atmosphere (Beaulieu et al., 2011; Raymond et al., 2013; Yao et al., 2020). Concentrations of CO_2_ and N_2_O declined with polynomial functions with increasing distance from headwaters along the Gwynns Falls (Figure 6). Biogeochemical processing of excess organic matter and nutrients along urban watershed flowpaths can significantly enhance production of GHGs (Smith et al., 2017; Jin et al., 2018). However, excess organic matter may also accumulate along urban watershed continuum flowpaths and become more labile and reactive (Kaushal and Belt, 2012; Kaushal et al., 2014a) when organic matter supply exceeds biological demand. Sewage inputs can also contribute to increased GHG concentrations along flowpaths (Jin et al., 2018), and sewage leaks are prevalent along the Gwynns Falls watershed (Kaushal et al., 2011). Concentrations of GHGs along the urban watershed continuum are influenced by other complex factors such as relationships of algal blooms, gas concentrations with discharge, gas exchange with the atmosphere, turbulence and seasonal hydrology (Harrison et al., 2005; Raymond et al., 2012; Grant et al., 2018). Overall, LSS monitoring of carbon, nutrients, and greenhouse gasses revealed coupled biogeochemical cycles along flowpaths and the potential for organic carbon overloads to downstream ecosystems (*sensu* Mallin et al., 2006; Blaszczak et al., 2019a; Blaszczak et al., 2019b; Carter et al., 2021).

#### Transformations of metals and salts along flowpaths –

The potential for water quality transformations along flowpaths is not just limited to carbon and nutrients, but also includes transformations in forms and types of metal and salts through biogeochemical processes. Excess anthropogenic salt ion inputs shift the natural chemistry of freshwaters and pose emerging risks for drinking water supplies, aquatic ecosystems, and infrastructure both regionally and globally (*e.g.*, Kaushal et al., 2005, Cañedo-Argüelles et al., 2013, Cañedo-Argüelles et al., 2016, Kaushal et al., 2020; Kaushal et al., 2022; Kaushal et al., 2023b; Bhide et al., 2021, Hintz et al., 2022b, Grant et al., 2022). We observed longitudinal variations in transport of both metals and salts along flowpaths. As expected, there were increasing trends in salts and metals with distance downstream along flowpaths draining progressively urban areas like the Gwynns Falls (Figure 4). These increasing concentrations were likely due to impervious surfaces and storm drains transporting metals and salts from pollution sources such as vehicles, tires, atmospheric deposition, road salts, sewage, *etc.* (Paul and Meyer, 2001; Kaushal et al., 2020; Morel et al., 2020) as watershed urbanization increased with distance downstream.

Salts and metals can increase or decrease along longitudinal flowpaths due to geochemical transformations, even over relatively small spatial scales along watersheds (Driscoll et al., 1988; Kaushal et al., 2022). Longitudinal patterns of conductivity (an indicator of salinity) and metals showed similar downstream decreasing patterns along Bull Run, Rock Creek, and Scotts Level Branch (Figure 10). Complexation of metals with salt ions, organic compounds, and inorganic materials can affect metal transport and transformation along flowpaths (Kaushal et al., 2020). Chloro-complexation between chloride ions and some metals could explain some similar patterns between conductivity and metals (Kaushal et al., 2019; Galella et al., 2021). For example, Cu can become soluble in response to increased salinity due to chloro-complexation (Kaushal et al., 2019). Ion exchange can also be important in explaining synoptic-scale patterns of salts and metals. Ion exchange retains ~30–40% of incoming Na on exchange sites in sediments of Campus Creek (Kaushal et al., 2022). Sodium ions can mobilize other base cations and metal ions by directly competing for binding sites on sediment and ion exchange reactions (Kaushal et al., 2019; Kaushal et al., 2022). Thus, co-mobilization of salt ions and metals can occur along urban flowpaths (Kaushal et al., 2019; Kaushal et al., 2020; Galella et al., 2021; Kaushal et al., 2022; Kaushal et al., 2023b; Kaushal et al., 2023).

### 4.4 Potential for tracking chemical cocktails and contributions from point and non-point sources

Principal components analysis of multiple elements along longitudinal flowpaths showed the potential to track fate and transport of pollution sources. For example, principal components analysis revealed a shift from labile protein-rich organic matter to more recalcitrant humic-like fractions of DOM as Bull Run flowed from a wastewater treatment plant through a forested regional park (Figure 12). Along the flowpath of Bull Run, principal components analysis also showed how the water quality signature of sewage effluent (enriched in K, Ca, S) from the UOSA wastewater treatment plant shifted to a natural groundwater signature enriched in Mg from geologic sources along downstream reaches in the forested regional park (Figure 13). These examples suggest LSS monitoring can be a powerful approach for tracking pulses and persistence of organic pollution inputs along streams (and also characterizing lag times in transformation of chemical mixtures). Future LSS approaches may also consider characterizing endmembers of different pollution sources, and then using endmembers to quantify contributions from different pollution sources along flowpaths using mixing models. Thus, LSS monitoring may provide a semiquantitative or quantitative approach for identifying and tracking the fate and transport of different pollution sources along streams and rivers.

### 4.5 Tracking attenuation of chemical cocktails in concert along conservation and restoration zones

Overall, LSS monitoring revealed the potential for attenuation and dilution of multiple chemical cocktails from wastewater, urban stormwater, and road salt. For example, there were rapid rates of chemical attenuation directly downstream of the UOSA wastewater treatment plant along the Bull Run flowpath through forested regional parks (Figure 10). This attenuation affected nutrients, organic matter, salts, and metals. There was rapid attenuation likely due to dilution and transport distances that were chemical/ element specific likely due to their propensity for biological uptake, chromatographic ion exchange in sediments and soils, sorption, sedimentation, storage, etc. along flowpaths (*e.g.*, Kaushal et al., 2018; Parker et al., 2021). There were also rapid transitions in downstream concentrations of chemical cocktails, as streamwater flowed through restored stream-floodplain complexes along Scotts Level Branch (Figure 10). There was attenuation and dilution of salt ions during spring and summer months along the Anacostia River flowpath as the river flowed through Anacostia National Park (Figure 7). There were also inverse relationships between some chemical concentrations and increasing % forest and shrub cover at the watershed scale along Bull Run, Rock Creek, and Scotts Level Branch flowpaths (Figure 11).

These attenuation processes and transitions could have sometimes been due to dilution in stream reaches draining into conservation areas in parks, which may have minimal inputs of pollution compared to upstream. In addition, there could have been elemental transformations of nutrients and metals from dissolved to particulate forms due to biological uptake and transformation to biomass, changes in pH and solubility, and deposition of particulates out of the water column (Driscoll et al., 1988; Marti et al., 2004; Haggard et al., 2005). There could have also been transient storage or reactive uptake of nutrients, salts, metals and organic matter in hyporheic zones, floodplain soils, and hydrologically connected wetlands in conservation and restoration areas (Butturini and Sabater, 1999; Fuller and Harvey, 2000; Kaushal et al., 2022). In addition, increased availability of natural organic matter could also have contributed to enhanced attenuation of nutrients and metals. For example, we observed distinct changes in the quality of organic matter along the Bull Run flowpath using fluorescence spectroscopy (Figure 12). Organic matter can influence denitrification and complexation between organic materials and metals (Groffman et al., 2005; Mayer et al., 2010; Kaushal et al., 2018). Nonetheless, the presence of forest cover, conservation areas, and parks may have the potential to influence localized water quality along stream reaches (Figure 11).

Surprisingly, we also observed some capacity for attenuating and diluting salt ions (both processes can be important) along flowpaths in conservation and restoration zones (Maas et al., 2021) (Figures 4,7,9,10). Freshwater Salinization Syndrome (FSS) from anthropogenic sources is altering the natural chemistry of freshwaters on local, regional, and global scales. FSS poses emerging risks for drinking water supplies, aquatic ecosystems, and infrastructure globally (Kaushal et al., 2005; Cañedo-Argüelles et al., 2013; Cañedo-Argüelles et al., 2016; Dugan et al., 2017; Kaushal et al., 2018; Hintz and Relyea, 2019; Oswald et al., 2019; Kaushal et al., 2020; Hintz et al., 2022b; Grant et al., 2022; Kaushal et al., 2022). Increased concentrations of salts at many of our sites were in ranges that could impair ecosystems and mobilize metals and other contaminants (Kaushal et al., 2019; Galella et al., 2021). There were decreasing concentrations of salt ions along Bull Run with increasing distance downstream from urban areas and wastewater treatment plant discharges (Figure 10). In addition, there was a plateau in concentrations of salt ions along Rock Creek, as the stream flowed from progressively urban areas into forested reaches of Rock Creek National Park (Figure 10). The presence of forest cover, conservation areas, and parks at the watershed scale may have the potential to attenuate salt pollution (Figure 11), but more work is necessary to assess limitations (Maas, 2022).

#### 4.5.1 LSS monitoring can help evaluate restoration efforts along the UWC

Although underappreciated, LSS monitoring of multiple contaminants has the potential to comprehensively examine the cumulative impacts of conservation and restoration along the entire stream flowpath from headwaters to receiving waters by measuring water quality changes due to source reductions, contaminant retention, and transformation. Overall, LSS monitoring showed that patterns in N, Fe, Mn, C, P, and dissolved oxygen, reveal trends and transitions in redox reactions and biogeochemical hot spots along flowpaths (*e.g.*, Marti et al., 2004; Haggard et al., 2005; Newcomer Johnson et al., 2014; Kaushal et al., 2014a; Ledford and Lautz, 2015). Concentrations of dissolved Fe and Mn may be tracers of groundwater inputs along restored streams with low dissolved oxygen and indicate the degree to hydrological connectivity between streams and floodplains and fringing wetlands (Figures 4, 7, 9). Furthermore, the chemical changes with distance downstream of various elements, for example, the reduction in nitrate per unit length of stream channel, could be quantified and used as a metric for assessing the efficacy of restoration and management and the subsequent recovery of systems. The advantage of examining pairs of chemicals, as just one example the inverse relationship between nitrate and dissolved organic carbon or different ions involved in ion exchange reactions, can elucidate the mechanisms behind the pattern. In addition, the role of physical dilution as a driver can be explored, if slopes between sampling points are roughly equal across conservative parameters (conductivity) or a wide variety of analytes. Finally, monitoring the suite of chemicals (chemical cocktails) along with ancillary factors (pH, redox, conductivity, temperature) will further reveal mechanisms behind synoptic patterns and provide a more substantial basis for establishing best management practices for pollutants of concern.

#### 4.5.2 Using LSS monitoring approaches in water quality studies: Future considerations

Ultimately, there are both advantages and challenges in using LSS monitoring approaches in water quality studies. A primary advantage of LSS monitoring can be the explicit integration of space and time in the monitoring design. LSS monitoring combined with routine monitoring over time can be effective because it provides comprehensive tracking of sources and sinks of contaminants across space and time (especially when multiple contaminants are analyzed together using a chemical cocktail approach). LSS monitoring provides high spatial resolution assessment of water quality in heterogeneous urban landscapes. Management interventions are often located in discrete segments throughout a watershed and implemented at a stream reach scale based on project and goals (Kaushal et al., 2023a). Monitoring along an entire watershed flowpath provides critical contextual information relevant to: (1) evaluating effectiveness of specific restoration features, (2) determining how far the restoration signal persists downstream, (3) quantifying rates of changes in concentrations and loads with distance downstream, watershed area, forest cover, or impervious surface cover, and (4) tracking sources and sinks of combinations of chemical contaminants to identify tradeoffs or the potential for co-management of chemicals (Kaushal et al., 2023a). LSS monitoring can also yield insights into prioritizing where future restoration efforts may be most successful (e.g. degraded stream reaches where rates of changes in chemical concentrations or loads are most likely to occur). Analysis of multiple chemicals together can reveal water quality tradeoffs (such as dissolved oxygen concentrations dropping to almost 0 mg/L in regenerative stormwater conveyance restoration features designed to promote denitrification) (Figure 9). Information from multiple chemicals can also be used in developing source tracking tools for understanding the fate and transport of non-point pollution sources along flowpaths using multivariate statistics.

There are also potential challenges associated with LSS monitoring such as making comparisons across baseflow (steady-state conditions) vs. storms, and the use of appropriate statistical approaches when analyzing data. In some cases, LSS monitoring may occur relatively infrequently (maybe only during a few seasons) and not always at the same fixed sampling stations over time. There may be a need to analyze LSS monitoring data across baseflow and stormflow conditions separately. Conducting LSS monitoring more routinely at fixed locations along the stream could help with discerning water quality patterns across both space and time. Dealing with spatial and temporal autocorrelation may also present a challenge for analyzing LSS monitoring data. Analytical approaches may need to be considered in a multivariate context with consideration given to multiple response variables and multiple predictors as well as non-independence of the sampling locations. However, there are also many spatial statistical and geospatial approaches such as variograms, semivariograms, and kriging for analyzing downstream patterns in water quality. In order to estimate transition distances and spatial lags in concentrations and loads along flowpaths, it may be possible to analyze spatial trends along stream reaches and detect monotonic changes with distance downstream for detecting transition zones (Hintz et al., 2022a). There also may be multivariate approaches to analyze changes in chemical cocktails, or distinct groups of elemental analytes (Mg, Fe, Mn, Ba, Na, Ca, S, K, Cu, *etc.*) along flowpaths (Figures 12, 13), to identify patterns in chemical analytes along flowpaths. Much time and effort has focused on analyzing temporal trends in water quality. However, visualizing, illustrating, and statistically analyzing and comparing longitudinal trends and transitions across watersheds represents a frontier in hydrologic sciences, water quality monitoring, and watershed biogeochemistry.

## 5 Conclusion

There is a growing need for understanding the transport and transformation of chemical cocktails along watershed flowpaths to protect and restore urban water quality. In particular, PCAs and other multivariate interpretations and modeling approaches can have considerable impact when applying synoptic-scale monitoring (particularly, when analytes are considered in concert as chemical cocktails rather than in isolation). A LSS monitoring approach can facilitate addition of new locations over time, or it can be along fixed locations. There have not been as many LSS studies compared with other monitoring approaches, but despite this, we have formed a framework. Although there are advantages and challenges, LSS monitoring can provide novel results for multiple contaminants at the relevant watershed scales with implications for receiving waters. LSS monitoring has potential to quantify chemical loads and analyze how they change along flowpaths, particularly during baseflow and steady state conditions. LSS monitoring can quantify changes in loads for multiple combinations of chemicals (nutrients, metals, and salts), and we can analyze whether changes in loads occur together or show divergent patterns along different watersheds. If studies only monitor a few locations in heterogeneous and human-impacted landscapes, critical information on sources, transport, and transformations of pollutants may be missing along flowpaths to receiving waters, particularly in urban watersheds (Kaushal and Belt, 2012; Kaushal et al., 2014a; Kaushal et al., 2023a). There is a particularly strong need to apply LSS monitoring approaches, where there can be considerable reach-scale heterogeneity in land use, lithology, and management surrounding streams (Kaushal et al., 2023a). Our results suggest that a LSS monitoring approach: (1) can reveal detailed water quality trends and transitions across expanding spatial scales, land use and management, geology and hydrology; (2) can evaluate effects of watershed management beyond stream reach scales; and (4) can track and compare rates of change per distance downstream in the sources, transport, and transformations of chemical cocktails *en route* to sensitive receiving waters such as drinking water supplies, reservoirs, and coastal zones.

## Supplementary Material

Supplement1

## Figures and Tables

**TABLE 1:** Some examples of longitudinal typologies of water quality responses along streams and rivers from the global literature.

Longitudinal water quality typologies	Location	Land use/Inputs	Longitudinal patterns in chemical concentrations along flowpaths	Longitudinal processes along flowpaths	Literature reference
1. Increasing typology					
Willipa and North Rivers	Washington, United States	Forest to Agriculture	Increase in SRP and turbidity with distance downstream	Surrounding land use impacts longitudinal water quality	Gove et al. (2001)
Bagmati River	Kathmandu, Nepal	Forest to Urban	Increase in Cl^−^, PO_4_^3−^, DOC, TDN, NH_4_^+^, Na, K, Mg, Ca, SiO_2_ with distance downstream	Urbanization increases multiple chemical cocktails	Bhatt and McDowell (2007)
Upper Mud River	West Virginia, United States of America	Mining to Forest	Increase in Se, SO_4_^2−^, conductivity, and other elements with distance downstream	Mining accelerates weathering reactions	Lindberg et al., (2011)
Hubbard Brook	New Hampshire, United States	Forest	Increases in pH, Ca, and Na along tributaries	Acid neutralization and buffering increase along flowpaths	Likens and Buso (2006)
Gwynns Falls	Maryland, United States	Suburban to Urban	Increases in Mg, Ca, DIC, and pH with distance downstream along an urban stream	Increasing watershed impervious surface cover and human-accelerated weathering	Kaushal et al., (2017)
2. Decreasing/Dilution Typology					
15 Catalonia Streams	Spain	Wastewater Inputs	Decrease and dilution of DIN and phosphate	Nutrient uptake saturation in streams from wastewater	Marti et al., (2004)
Dead Run	Maryland, United States	Urban	Decrease and dilution of chloride and sulfate for part of flowpath downstream from road	Spatial heterogeneity of non-point sources and legacy groundwater contamination	Welty et al., (2023)
3. Chemostatic Typology					
Gibson Jack Stream	Idaho, United States	Forest, shrub, grass	Chemostasis - variability in DOC concentrations decreased with watershed size	Steady state concentrations with groundwater and surface water	Hale and Godsey (2019)
4. Transition Zones Typology					
Meadowbrook Creek	New York, United States of America	Suburban	Transition zone and increase in Cl^−^ and NO_3_^−^ in restored stream	Hydrologic storage of chemicals in reconnected floodplain	Ledford and Lautz (2015)
Minebank Run	Maryland, United States	Urban	Transition zone as salt ions increased abruptly downstream of major roadway	Salinization increased from roadway and ion exchange	Cooper et al., (2014)
5. Pulses Typology					
Dead Run	Maryland, United States	Urban	Pulses in nitrate isotopes with distance downstream	Spatial heterogeneity of nonpoint nitrate sources	Pennino et al., (2016b)
Han River	South Korea	Urban	Pulses in N_2_O and CO_2_ increase rapidly after wastewater treatment discharges	Hot spots of microbial activity/ coupled cycles	Jin et al., (2018)
Spring Branch, Minebank Run, Powder Mill Run, Dead Run	Maryland, United States	Urban	Pulses in concentrations of Ca and Mg	Pulses downstream of road and bridge crossings	Sivirichi et al., (2011)
Upper Mississippi River	Upper Midwest, United States	Agriculture	Pulses in specific conductivity	Tributary inputs	Loken et al., (2018)
6. Plateaus Typology					
Upper Mississippi River	Upper Midwest, United States	Agriculture	Plateau in nitrate concentrations after saturation of biogeochemical uptake with distance downstream	N saturation of in-stream biological demand	Loken et al., (2018)
15 Catalonia Streams	Spain	Wastewater Inputs	Plateau in biological nutrient uptake	Nutrient uptake saturation in streams from wastewater	Marti et al., (2004)
Meadowbrook Creek	New York, United States	Suburban	Plateaus in NO_3_^−^ and SO_4_^2−^	Plateaus occurred downstream of transition zone along a restored stream	Ledford and Lautz (2015)
7. Biogeochemical Uptake Typology					
Spring Branch, Gwynns Run, Pond Branch	Maryland, United States	Urban	Biogeochemical uptake and decrease in TDN through restored floodplain/wetlands and stream-wetland complexes	Restoration enhances N uptake and denitrification	Newcomer Johnson et al., (2014)
Gwynns Falls	Maryland, United States	Suburban to Urban	Biogeochemical uptake and transformation - Decreasing TDN and increasing DOC downstream	Urban streams are transformers with high N uptake, denitrification, DOC production	Kaushal et al., (2014a)
6 spring-fed rivers in North Florida	Florida, United States of America	Spring-fed rivers - mixed land use - forest, agriculture, and urban	Chemostatic input of N creates steady-state nutrient enrichment experiment and biogeochemical uptake contributed to exponential and linear decreases in NO_3_^−^	Greater uptake in heavily vegetated reaches with lower specific discharge	Hensley et al., (2014)
8. Transformation Typology					
Columbia Hollow	Arkansas, United States	Wastewater Inputs	Transformation: downstream decrease in NH_4_^+^ and increase in nitrate and TDN	Nitrification downstream of wastewater discharges	Haggard et al., (2005)
Spring Branch, Minebank Run, Powder Mill Run, Dead Run	Maryland, United States	Urban	Transformation - decreasing TDN and increasing DOC downstream	Restoration enhances N uptake and denitrification	Sivirichi et al., (2011)
Dead Run and Red Run	Maryland, United States	Urban	Transformation: downstream relationships between TDN and DOC and N_2_O, CO_2_, and CH_4_	Urban streams are sources of N_2_O, CO_2_, and CH_4_	Smith et al., (2017)
Campus Creek	Maryland, United States	Urban	Transformation: decrease in Na and increase in P with distance downstream	Ion exchange and P mobilization from road salt	Kaushal et al.,(2023b)
North Branch of the Moose River	New York, United State	Forest	Transformation: Changes in soluble vs. particulate Al. Increases in acid neutralizing capacity and decreases in soluble Al with distance downstream	Acid neutralization and buffering increase along flowpaths	Driscoll et al., (1988)
9. Complex Piecewise Typologies and Transitions in Water Quality Trends for Different Stream Segments					

Many studies can show complex piecewise trends when aggregating study reaches or multiple typologies along flowpaths, and different chemicals can show different longitudinal typologies due to different processes. Therefore, it may be best to break up longitudinal water quality patterns along streams and rivers into piecewise functions for segments reflecting changes in land use, geology, and management

**TABLE 2:** Information regarding longitudinal stream synoptic (LSS) monitoring surveys along different watersheds in the Chesapeake Bay region. Ten watershed flowpaths were analyzed. Within the Anacostia watershed, we studied 5 streams: Sligo Creek, Campus Creek, Paint Branch, Northeast Branch, and Northwest B. The other 5 streams (Bull Run, Gwynns Falls, Rock Creek, and Scotts Level Branch) are monitored by the United States Geological Survey (USGS) and/or other entities.

Stream	Sampling dates	Longitudinal distance sampled (km)	Number of samples collecte			USGS station number	Discharge at USGS station (m^3^/s)
			*Mainstem*	*Median distance between mainstem sampling sites (km)*	*Tributary*		
*Anacostia*	3–4, 6 May, 2012	24.7	20	0.7	15	01651007	
	17 January 2022	9.0	12	0.7	0		
	13 April 2022	10.5	14	0.7	0		
	10 May 2022	10.5	14	0.7	0		
	18 June 2022	10.5	14	0.7	0		
	14 July 2022	10.5	14	0.7	0		
	15 August 2022	10.5	14	0.7	0		
*Northeast Branch*	May 3–4, 6, 2012	3.4	4		0	01649500	0.75
*Paint Branch*	May 3–4, 6, 2012	4.5	4	1.3	0	01649190	0.19
*Campus Creek*	3 February 2021	1.2	11	0.1	0		
*Northwest Branch*	12 February 2021	1.2	11	0.1	0		
	19 February 2021	1.2	11	0.1	0		
	5 July 2022	1.3	12	0.1	0		
	15 July 2022	1.3	12	0.1	0		
	19 July 2022	1.3	12	0.1	0		
	2 August 2022	1.3	12	0.1	0		
	9 August 2022	1.3	12	0.1	0		
	16 August 2022	1.3	12	0.1	0		
	23 August 2022	1.3	12	0.1	0		
	31 August 2022	1.3	12	0.1	0		
	14 September 2022	1.3	12	0.1	0		
	26 September 2022	1.3	12	0.1	0		
	May 3–4, 6, 2012	12.1	11	1.1	0	01651000	0.42
*Sligo Creek*	May 3–4, 6, 2012	13.1	11	1.3	0	01650800	0.05
*Bull Run*	13 January 2021	16.4	25	0.5	18		
	17 September 2021	16.8	42	0.2	23		
	08 January 2022	2.47	15	0.2	4		
	17 January 2022	2.50	15	0.2	5		
*Gwynns Falls*	24 September 2008	18.5	3	18.3	2		0.54
	October 27–31, 2008	36.5	21	1.4	25		0.88
	16 December 2008	36.5	12	4.5	8	01589352	1.61
	January 13–14, 2009	30.6	13	1.7	18		1.18
*Rock Creek*	6 February 2021	21.2	18	1.2	6		1.49
	13 August 2021	32.3	16	1.0	4	01648010	0.82
	19 January 2022	20.9	17	0.6	8		2.02
*Scotts Level Branch*	27 March 2021	8.6	12	0.8	0		0.06
	12 August 2021	8.6	12	0.8	0		0.01
	2 September 2021	8.6	12	0.8	0	01589290	0.04
	5 January 2022	8.6	12	0.8	0		0.03
	12 January 2022	8.6	13	0.7	0		0.03

## Data Availability

The raw data supporting the conclusions of this article will be made available by the authors, without undue reservation.
