# Supplementary material for: Longitudinal stream synoptic monitoring tracks chemicals along watershed continuums: a typology of trends
Source: Front Environ Sci. Author manuscript; Available in PMC 2024 Jun 9. (PMC10355011; doi:10.3389/fenvs.2023.1122485)
Supplement: Supplement1 [file NIHMS1908812-supplement-Supplement1.pdf]

Supplemental Information

Study Site	Latitude and Longitude at outflow	Watershed Area (km^2)	Watershed Forest Cover (%)	Watershed Impervious Surface Cover (%)	Watershed Urban Development (%)
Anacostia	38.86106, -77.01287	448.1	20.7	35.3	72.2
Northeast Branch	38.94301, -76.94391	194.3	27.5	29	63
Paint Branch	38.97869, -76.91741	80.8	25.2	31.9	67.8
Campus Creek	38.99282, -76.93556	1.8	22.1	26.9	77.5
Northwest Branch	38.94316, -76.94435	135.5	19.9	33.5	71.5
Sligo Creek	38.98121, -76.99094	19.8	12.2	41.6	27.2
Bull Run	38.72412, -77.38027	502.5	35.7	13	44.3
Gwynns Falls	39.26859, -76.62651	173.3	17.8	42.3	79.9
Rock Creek	38.90008, -77.05738	198.1	20.4	32.4	71.4
Scotts Level Branch	39.36058, -76.74620	10.4	13.2	39.3	83.8

Table 1. Land use characteristics within each study sites watershed. The watershed urban development was from the NLDC 2011 classes 21-24. The watershed characteristics were obtained using StreamStats at the latitude and longitude at the outfall (U.S. Geological Survey, 2016; National Land Cover Database 2011 (NLCD2011) Legend).

Longitudinal and lateral sampling of water quality along the UWC

Multiple longitudinal synoptic sampling locations were located throughout each of the above watersheds to investigate longitudinal changes in downstream concentrations and/or isotopic compositions of multiple chemical contaminants. In addition, we also sampled shallow groundwater at Scotts Level Branch along the flowpath from uplands to the stream channel using transects of piezometers. Methods of sampling for groundwater at Scotts Level Branch and details on groundwater well installation are in Wood et al. (2022). In each watershed, LSS monitoring was completed within 24 hours or on consecutive days with similar streamflow conditions. We did not always move downstream along a watershed during the day; in some cases, we moved upstream or sampled the entire watershed flowpath by multiple teams simultaneously throughout the day (*e.g.*, sampling timing was sometimes different for flowpaths and data collection could sometimes rotate through upstream, downstream, and simultaneous collection along flowpaths). Thus, the chemical patterns along flowpaths were not due to diurnal patterns throughout the day. Our sampling resolution between sampling points varied based on the size of the watershed, but was typically hundreds of meters or a few kilometers; thus, we sampled in detail along the length of each watershed to evaluate where urban degradation occurred and/or restoration activities were implemented. In some cases, these flowpaths extended from headwaters to the stream outflow to receiving waters. Sampling locations for the synoptic sites along each mainstem were chosen based on accessibility, presence of tributary junctions, and positioning of conservation and restoration features.

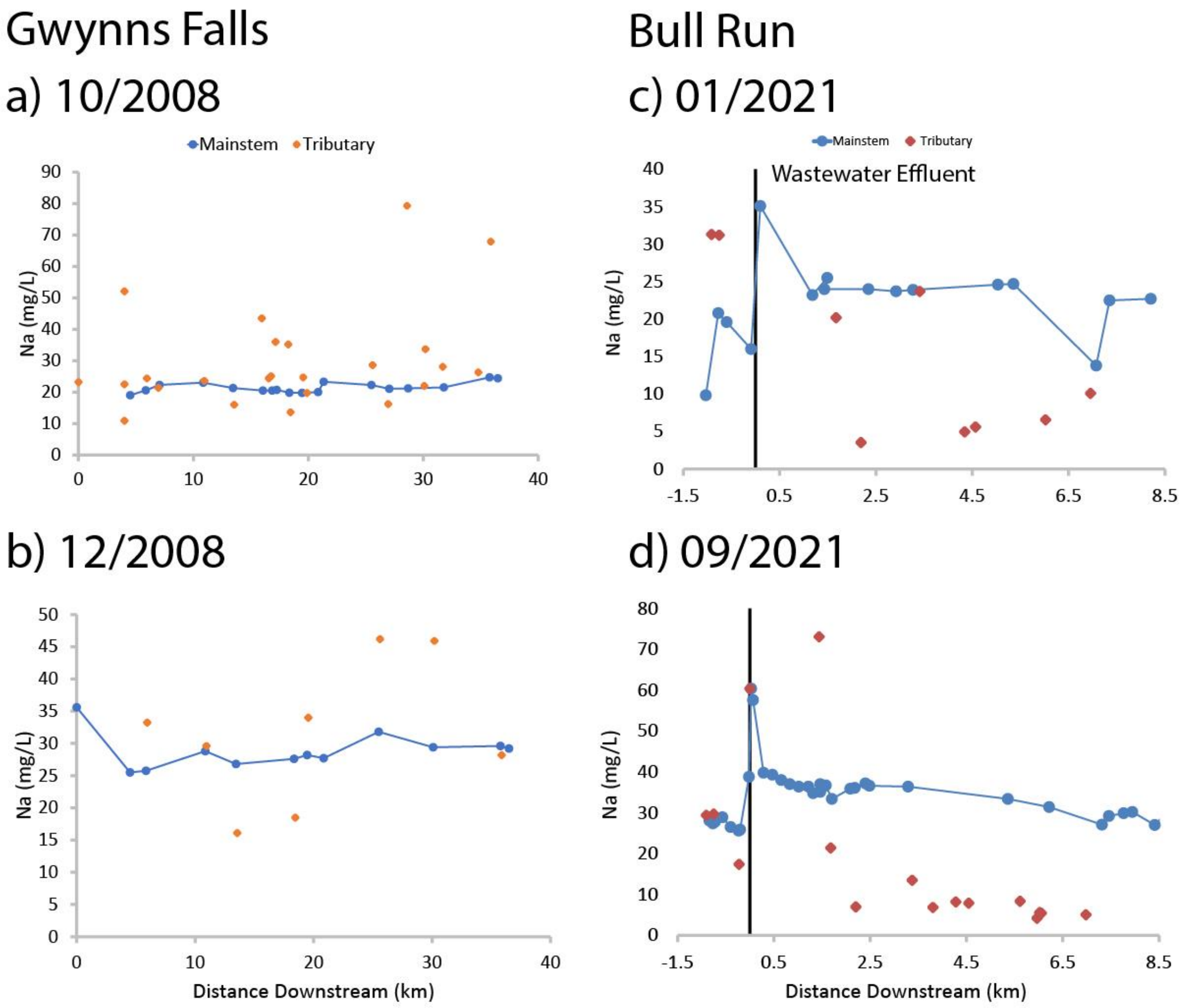

Supplemental Figure 1. Changes in Na concentrations along the Gwynns Falls mainstem are not substantially

Figure Number	Parameter	Equation	R^2	p-values
4a	Ca	$y = 0.014x^2 - 0.21x + 26.5$	0.78	$3.5x10^{-6}$
	Mg	$y = 0.0052x^2 - 0.096x + 10.7$	0.51	0.001
	S	$y = 0.0031x^2 - 0.015x + 2.35$	0.92	$1.7x10^{-9}$
	B	$y = 0.0046e^{0.0398x}$	0.89	$1.1x10^{-8}$
	Mo	$y = 3x10^{-5}x + 0.0054$	0.23	0.028
4b	TDN	$y = 0.0021x^2 - 0.11x + 2.37$	0.51	0.011
	Ba	$y = -0.0011x + 0.0727$	0.73	$7.21x10^{-7}$
	Mn	$y = -0.0007x + 0.0439$	0.30	0.010
4d	Ca	$y = 0.39x^2 - 4.23x + 50.3$	0.80	0.027
	Mg	$y = 0.56x^2 - 6.22x + 25.45$	0.90	0.011
	Ba	$y = -0.0104x + 0.16$	0.71	0.001
4e	Fe	$y = -0.0054x^2 + 0.0502x + 0.045$	0.79	0.32
	Mn	$y = -0.0013x^3 + 0.018x^2 - 0.073x + 0.12$	0.48	0.11
4f	K: After restoration	$y = 0.18x + 1.12$	0.99	0.053
	K: After Park	$y = -0.14x + 2.39$	0.93	0.002
	Sr: Upstream	$y = -0.0024x + 0.16$	0.79	0.000
5	TDN	$y = 2.98x^2 - 4x + 2.18$	0.86	0.00016
	Ba	$y = -0.031x + 0.066$	0.75	$7.81x10^{-6}$
	S	$y = 2.99x + 1.9$	0.52	0.0011
	B	$y = 0.03x^2 + 0.012x + 0.0075$	0.77	$7.1x10^{-5}$
6	N_2O	$y = 7x10^{-5}x^2 - 0.0039x + 0.068$	0.64	$8.0x10^{-5}$
	CO_2	$y = 0.37x^2 - 19.54x + 272.45$	0.50	0.001
	DOC	$y = 8x10^{-5}x^4 - 0.0068x^3 + 0.19x^2 - 1.64x + 5.62$	0.59	0.087
	HIX	$y = 0.0001x^2 - 0.0056x + 0.8557$	0.45	0.020
7: Na	01/17/22	$y = 2.99x + 204.95$	0.42	0.023
	04/13/22	$y = -1.50x + 49.42$	0.56	0.0021
	05/10/22	$y = -1.59x + 32.76$	0.80	$1.68x10^{-5}$
	06/18/22	$y = -1.90x + 38.26$	0.96	$5.31x10^{-10}$
	08/15/22	$y = -1.54x + 24.87$	0.74	$8.06x10^{-5}$
7: K	04/13/22	$y = -0.070 + 3.81$	0.41	$1.33x10^{-2}$
	08/15/22	$y = -0.10 + 3.91$	0.67	$3.32x10^{-4}$
9: Mn	7/5/2022	$y = 0.0141x^2 - 0.6213x + 7.0155$	0.1171	
	7/15/2022	$y = 0.0585x^2 – 2.523x + 27.286$	0.2322	
	7/19/2022	$y = 0.0391x^2 – 1.5889x +16.395$	0.556	
	8/2/2022	$y = 0.0636x^2 – 2.7465x + 29.653$	0.7908	
	8/9/2022	$y = 0.0502x^2 – 2.1435x +23.005$	0.449	
	8/16/2022	$y = 0.0411x^2 – 1.7638x +18.999$	0.68	
	8/23/2022	$y = 0.0164x^2 – 0.7269x +8.1688$	0.337	
	8/31/2022	$y = 0.059x^2 – 2.5754x +28.041$	0.688	
	9/14/2022	$y = 0.0293x^2 – 1.2408x + 13.186$	0.393	
	9/26/2022	$y = 0.0246x^2 – 1.0348x + 10.902$	0.320	
9: Fe	7/5/2022	$y = 0.0209x^2 - 0.95x + 11.428$	0.2283	
	7/15/2022	$y = 0.0244x^2 - 1.1393x + 13.417$	0.8173	
	7/19/2022	$y = 0.0464x^2 - 2.1349x + 25.166$	0.8358	
	8/2/2022	$y = 0.0894x^2 - 3.8135x + 41.024$	0.8136	
	8/9/2022	$y = 0.0266x^2 - 1.1045x + 12.297$	0.4064	

	8/16/2022<br>8/23/2022<br>8/31/2022<br>9/14/2022<br>9/26/2022	$y = -0.0074x^2 + 0.4402x - 5.3567$<br>$y = 0.0063x^2 - 0.2494x + 2.9941$<br>$y = 0.0315x^2 - 1.3581x + 15.006$<br>$y = 0.0276x^2 - 1.1284x + 12.157$<br>$y = 0.0235x^2 - 0.9984x + 10.766$	0.8587<br>0.5704<br>0.4081<br>0.6657<br>0.4191	
10d	Na<br>Ca<br>Mg	$y = 83.4x10^{-1.01}x$<br>$y = 3.15x^2 - 29.67x + 71$<br>$y = -3.58x + 14.64$	0.83<br>0.99<br>0.90	
13b	Na<br>Temperature<br>DIC	$y = 0.03x + 0.27$<br>$y = 1.42x + 1.38$<br>$y = 2.16x + 18.75$	0.48<br>0.89<br>0.44	$1.87\ x10^{-7}$<br>0.010<br>0.026
13d	BIX<br>FI<br>K	$y = 0.028x + 0.65$<br>$y = 0.054x + 1.45$<br>$y = -1.06x^2 + 9.33x - 12.32$	0.95<br>0.85<br>0.82	$5.26x10^{-11}$<br>$8.38x10^{-13}$<br>0.017

Supplemental Table 2. Equations, p values, and R^2 values for relationships shown in the figures.
